# Following Tetraploidy in Maize, a Short Deletion Mechanism Removed Genes Preferentially from One of the Two Homeologs

**DOI:** 10.1371/journal.pbio.1000409

**Published:** 2010-06-29

**Authors:** Margaret R. Woodhouse, James C. Schnable, Brent S. Pedersen, Eric Lyons, Damon Lisch, Shabarinath Subramaniam, Michael Freeling

**Affiliations:** Department of Plant and Microbial Biology, University of California Berkeley, Berkeley, California, United States of America; Trinity College Dublin, Ireland

## Abstract

Previous work in Arabidopsis showed that after an ancient tetraploidy event, genes were preferentially removed from one of the two homeologs, a process known as fractionation. The mechanism of fractionation is unknown. We sought to determine whether such preferential, or biased, fractionation exists in maize and, if so, whether a specific mechanism could be implicated in this process. We studied the process of fractionation using two recently sequenced grass species: sorghum and maize. The maize lineage has experienced a tetraploidy since its divergence from sorghum approximately 12 million years ago, and fragments of many knocked-out genes retain enough sequence similarity to be easily identifiable. Using sorghum exons as the query sequence, we studied the fate of both orthologous genes in maize following the maize tetraploidy. We show that genes are predominantly lost, not relocated, and that single-gene loss by deletion is the rule. Based on comparisons with orthologous sorghum and rice genes, we also infer that the sequences present before the deletion events were flanked by short direct repeats, a signature of intra-chromosomal recombination. Evidence of this deletion mechanism is found 2.3 times more frequently on one of the maize homeologs, consistent with earlier observations of biased fractionation. The over-fractionated homeolog is also a greater than 3-fold better target for transposon removal, but does not have an observably higher synonymous base substitution rate, nor could we find differentially placed methylation domains. We conclude that fractionation is indeed biased in maize and that intra-chromosomal or possibly a similar illegitimate recombination is the primary mechanism by which fractionation occurs. The mechanism of intra-chromosomal recombination explains the observed bias in both gene and transposon loss in the maize lineage. The existence of fractionation bias demonstrates that the frequency of deletion is modulated. Among the evolutionary benefits of this deletion/fractionation mechanism is bulk DNA removal and the generation of novel combinations of regulatory sequences and coding regions.

## Abstract 2

Following genome duplication and selfish DNA expansion, maize used a heretofore unknown mechanism to shed redundant genes and functionless DNA with bias toward one of the parental genomes.

## Author Summary

All genomes can accumulate dispensable DNA in the form of duplications of individual genes or even partial or whole genome duplications. Genomes also can accumulate selfish DNA elements. Duplication events specifically are often followed by extensive gene loss. The maize genome is particularly extreme, having become tetraploid 10 million years ago and played host to massive transposon amplifications. We compared the genome of sorghum (which is homologous to the pre-tetraploid maize genome) with the two identifiable parental genomes retained in maize. The two maize genomes differ greatly: one of the parental genomes has lost 2.3 times more genes than the other, and the selfish DNA regions between genes were even more frequently lost, suggesting maize can distinguish between the parental genomes present in the original tetraploid. We show that genes are actually lost, not simply relocated. Deletions were rarely longer than a single gene, and occurred between repeated DNA sequences, suggesting mis-recombination as a mechanism of gene removal. We hypothesize an epigenetic mechanism of genome distinction to account for the selective loss. To the extent that the rate of base substitutions tracks time, we neither support nor refute claims of maize allotetraploidy. Finally, we explain why it makes sense that purifying selection in mammals does not operate at all like the gene and genome deletion program we describe here.

## Introduction

**Figure 1:**
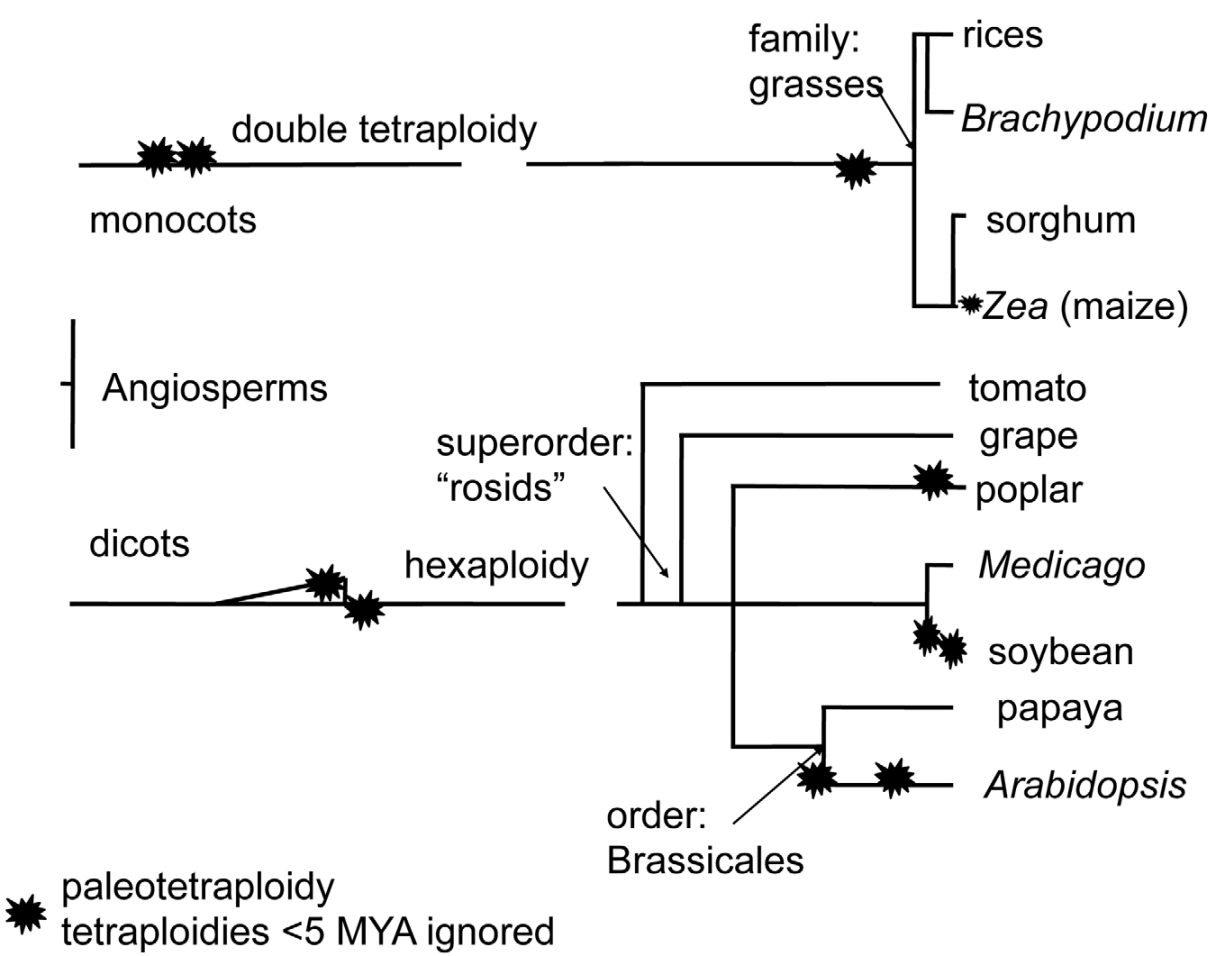
A heavily pruned phylogenetic tree of the sequenced genomes of flowering plants. Inferred, ancient (at least 5 million years old) tetraploidies are identified as stars. Citations are included in a recent review [19], except for the double tetraploidy at the base of the monocots [35] and the placement of the legume-specific tetraploidy (our tentative conclusion).

Decades ago it was proposed that whole-genome duplication provides raw material for evolutionary innovation, as reviewed [1]. The angiosperm phylogenetic tree of organisms with complete genome sequence has provided evidence for repeated ancient tetraploidies in all lineages (Figure 1). However, tetraploidies occurring before approximately 150 million years ago (MYA) in plants and 500 MYA in animals are difficult to detect [2]. Genomes that have experienced tetraploidy events tend to reduce their genome structure toward their ancestral chromosome number and gene content, though not gene order. The mutational process accomplishing this reduction in gene content is called fractionation, and its mechanism is unknown.

**Figure 2:**
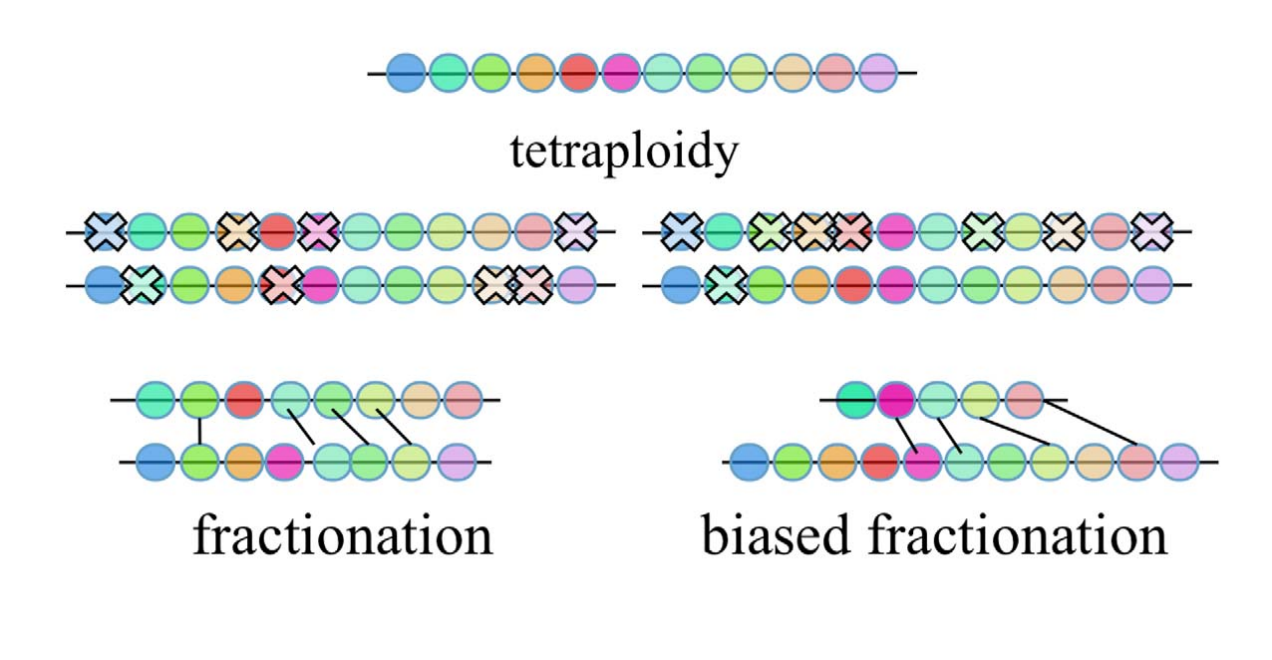
Cartoons illustrating random fractionation and biased fractionation. Lines connect homeologous genes retained as pairs, called “retained genes.” Note that retained genes cluster automatically after fractionation.

Theoretically, the expected fate of the average gene following tetraploidy is loss from one or the other, but not both, homeologous chromosomes [3],[4],. Previous studies on fractionation of the most recent tetraploidy in the Arabidopsis lineage (known as the alpha tetraploidy event) found significantly more gene loss on one homeolog than the other [7]. However, some genes are retained as homeologous pairs. This same study found that genes retained as pairs were significantly clustered and that *any* mechanism of fractionation causes clustering of retained genes, especially on the over-fractionated homeolog, as retained genes will inevitably be physically closer to each other once the intervening genes have been removed. Figure 2 illustrates expectations of biased and unbiased fractionation and shows how fractionation by any mechanism tends to cluster retained genes.

Any of the following gene loss mechanisms could contribute to fractionation after a tetraploidy event: (1) single gene loss via inactivation and sequence randomization (i.e. the pseudogene pathway) as observed in mammals, including primates [8]; (2) single gene “loss” from orthologous sites by gene transposition, as was observed in the Brassicales [9]; (3) single gene loss by a short deletion mechanism; (4) multiple gene loss events of any type, like long (multi-gene) deletions or segmental transpositions. Lai and coworkers [10] compared five orthologous panels of bacterial artificial chromosome sequence for rice, sorghum, maize homeolog 1, and maize homeolog 2. Each set of panels was anchored on a gene shared by all four genomes. They found examples of genes that moved out of the syntenic position in maize but were conserved syntenically between rice and sorghum.

Another likely mechanism for fractionation is short deletions via illegitimate, or intra-chromosomal, recombination, as introduced in point 3 above. Devos and coworkers [11] implicated recombination, both homologous and illegitimate, as the mechanism used by plants, including maize, to remove retrotransposons. This suggestion was based on finding short direct repeats from 2–13 bp, sometimes imperfect, flanking small deletions in the inferred target chromosome. This was the same conclusion derived previously from data implicating short deletions in nonfunctional transposons in Drosophila [12]. Citing bacterial illegitimate recombination studies, these researchers implicated recombination mechanisms as the transposon loss mechanism.

Using sorghum as our primary outgroup and rice as a secondary outgroup, we examine in detail the gene and chromosome fragments identifiable at the current stage of fractionation in the maize inbred B73, a genome sequenced recently [13]. We also examine such fragments in the recently sequenced soybean genome that result from a tetraploidy estimated to have occurred approximately 13 MYA [14]. We conclude that the most likely mechanism of fractionation is single gene loss by short deletions, predominantly in sizes ranging from 5 bp to 178 bp, with deletions being found 2.3 times more often on one homeolog than the other; infrequent longer deletions are possible. The fractionation mechanism, like the mechanism of transposon removal, is likely to be intra-chromosomal recombination, and this has general implications for bulk DNA removal and the wholesale generation of new sequence combinations.

## Results

### Manual Gene Retention and Fractionation Bias Data for 37 Panels of Orthologous Sorghum-Maize1-Maize2 Chromosomal Segments

To study the post-tetraploidy fractionation process in detail, both a sequenced genome that is undergoing fractionation and an outgroup with a sequenced genome that diverged before the tetraploidy event is required. For the most recent tetraploidy in maize, which happened from 5 to 12 MYA [15],[16], sorghum is such an outgroup. Sorghum diverged from the maize lineage just before the tetraploidy event (Figure 1) [17]. Sorghum has been sequenced [16], and the first assembly of a maize genome has recently been published [13]. In addition to its phylogenetic position, the maize lineage tetraploidy possesses another characteristic that recommends it for the study of fractionation: it is relatively recent. The alpha tetraploidy in Arabidopsis, at 23–50 MYA, is older than the most recent (alpha) tetraploidy in maize. Even so, the maize alpha tetraploidy is known to be highly fractionated [10],[18]. Our primary research aim was to detail what happened to those orthologous (syntenic) genes shared by sorghum and maize following the maize tetraploidy.

**Figure 3:**
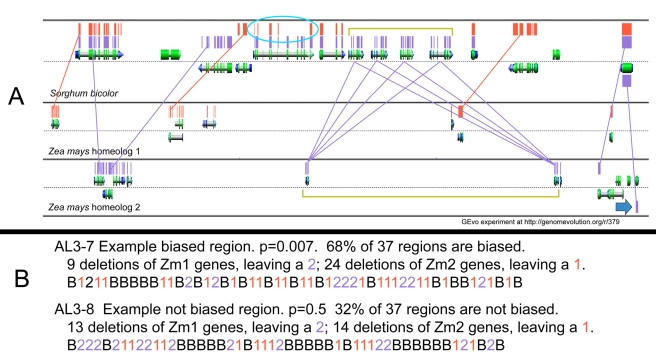
Determining which maize homeologous genes were deleted following tetraploidy. (A) Cartoon of a GEvo blastn comparison graphic depicting a 13-gene stretch of sorghum with the two orthologous regions of maize. Sorghum nucleotides were masked except those in official gene models or genes shared between sorghum and maize (SI1); maize DNA was masked for repeat sequences. Blastn high scoring pairs (hits) are colored orange if they are *Sb-Zm1* and purple if they are *Sb-Zm2*. Colored lines indicate orthology. The code B021BB2DDDB10B abbreviates these data, where B = both genes remain, 0 = gene missing in syntenous *Zm* positions (these are discarded), 1 = the gene on *Zm1* remains alone because its homeolog was deleted, 2 = the gene on *Zm2* remains alone because its homeolog was deleted, and D = local duplicates of an arbitrary mother gene leftmost in the cluster (D's are discarded). Therefore, the essential code for this 13-gene *Sb* stretch reduces to B21BB2B1B. The circle indicates a *Zm1* gene that has some completely and some partially deleted exons; we noted these partially fractionated genes for further research, but we counted them as present (B). The brackets enclose clusters of tandemly duplicated genes in both *Sb* and *Zm2*. The arrow indicates a single gene in *Sb* hitting a reverse tandem duplication in maize; maize genes like this one were counted as present. Please use http://genomevolution.org/r/37e to reproduce on-the-fly the *Sb2* region blast experiment, the region containing *Sb02g030760-Sb02g030950* drawn above. (B) Using the same color code of the panel above, these are two exemplary small regions from the total of 37 regions comprising our syntenic dataset. The regions exemplifying fractionation bias (AL3–7) and no bias (AL3–8) are color-coded in such a way that the number of gene deletions suffered by *Zm* homeolog 1 versus *Zm* homeolog 2 is easy to count. A 1∶1 (*p* = 0.5) ratio of these deletions computes to not biased. *p*≤0.1 is weak support and ≤0.05 is strong support that these deletions significantly deviate from this 1∶1 null hypothesis.

Using the procedures described in Methods, we identified 37 orthologous regions between sorghum and the corresponding maize homeologs retained after the maize alpha tetraploidy event. From these regions, we found that of the 2,943 sorghum-maize (*Sb-Zm*) syntenic shared genes that we studied, 43% of them were retained as homeologous pairs in maize. Note that we count as present any significant fragment of gene. If the maize tetraploidy behaved as other known tetraploidies in plants and microbes, retained genes should be enriched in those encoding transcription factors, as reviewed [19]. Indeed, the frequency of genes encoding transcription factors was 4.3 times greater among the retained genes as compared to the fractionated genes. Figure 3A is a cartoon of a GEvo output screenshot of a 13-gene segment of one of the 37 orthologous regions (region *Sb2*) between sorghum and its two maize homeologs, as described in Methods. The GEvo comparative sequence alignment tool output generated the original blastn output detailed in Methods. Supplemental Information 1 (Dataset S1) gives our primary data as inferred from analyses like that shown in Figure 3A. Dataset S2 shows how any one sorghum chromosomal region is orthologous to two maize regions, generating information essential to construct our sorghum-maize1-maize2 regions.

One way to measure fractionation bias is to first assume that gene loss involves one gene independently from any other gene, and then count the number of gene losses (deletions) on one homeolog as compared to the number of gene losses on the other homeolog. If fractionation were unbiased, this ratio is expected to be 1∶1. Other measures of fractionation include total number of genes or base pairs in an orthologous stretch, but counting deletions of shared genes is most direct, so we present this first. Figure 3B shows two representative diagrams of our data for shorter regions (Dataset S3 contains all 37 such diagrams with bias statistics) and indicates that fractionation has been significantly biased in 68% of our regions. Using data from nine representative longer sorghum regions (Table 1), we conclude that the over-fractionated chromosomes have 2.3 times as many deleted genes as do the under-fractionated chromosomes.

**Table 1:** The organization of fractionation and similar data based on the designation *Zm*-under-fractionated (u) homeolog and *Zm* over-fractionated homeolog (o).

A. Name Region of Nine Control Regions	B. *Zm Chromosomes Under-Fractionated(u), Over-Fractionated(o)*	C. *p* Value of Deletion Fractionation Bias	D. Deletions on *Zm*-Under (u)	E. Deletions on *Zm*-Over (o)	F. Retained Genes in Region (B)	G. Total *Sb-Zm* Shared Genes (Orthologous) in Region	H. Fractionation Bias in Deletion Units u/o	I. Fractionation bias in *Zm* Segment Length in Shared Genes, u/o	J. Fractionation Bias in Length of *Zm* Segment in Maize Genome .org Genes u/o	K. Fractionation Bias in *Zm* Segment Length in bps	L. Average Over-Fractionated Ks/Under-Fractionated Ks for All Chosen Pairs. Mean±SD, Median, # *Sb-Zm1,Zm2* Shared Genes Chosen	M. Methylation Peaks/Mb-o/u(Deng Lab, 2009).	N. Map Units -u/Map Units-o (Ahn and Tanksley 1993)
Sb1	Zm1,Zm9 u,o	0	17	47	105	169	0.36	1.2	1.5	1.8	0.92±0.34, 0.92, 266	9.6	0.9
Sb2	Zm7,Zm2 u,o	0.13	26	36	29	91	0.72	1.2	1.6	1.7	ND	ND	ND
SB3	Zm3,Zm8 u,o	0.1	19	29	45	93	0.66	1.2	1.5	3.1	ND	ND	1.1
Sb4	Zm5,Zm4 u,o	0	7	54	45	106	0.13	1.9	1.3	1.6	0.99±0.45, 0.95, 381	1.0	ND
Sb6	Zm2,Zm10u,o	0.03	34	53	81	168	0.64	1.2	1.7	2.2	1.04±0.54,0.97, 913	0.8	1.9
Sb7	Zm6,Zm4 u,o	0	14	44	38	96	0.32	1.6	ND	2.7	ND	ND	ND
Sb8	Zm1,Zm3 o = u	0.58	11	11	4	26	1.00	1.0	0.6 or 1.6	0.6 or 1.6	ND	ND	ND
Sb9	Zm6,Zm8 u,o	0	28	77	93	198	0.36	1.4	1.4	2.5	0.91±0.39, 0.85, 132	0.8	ND
Sb10	Zm5,Zm6 u,o	0	25	69	86	180	0.36	1.4	1.7	2.5	0.98±0.49,1.02,80	1.1	ND

When a feature is equally represented on both homeologs, then its under/over ratio will be approximately 1, but when a feature is biased with regard to fractionation, then the under/over ratio will differ significantly from 1. All data to the right of the empty column involve under/over ratios for some or all of the nine control regions. The fractionation bias ratios of Column H are less than 1 because the under-fractionated chromosome, while longer, has fewer deleted genes. Invert these fractions to compare with the greater than 1 ratios of Columns I, J, and K. ND = no data.

**Figure 4:**
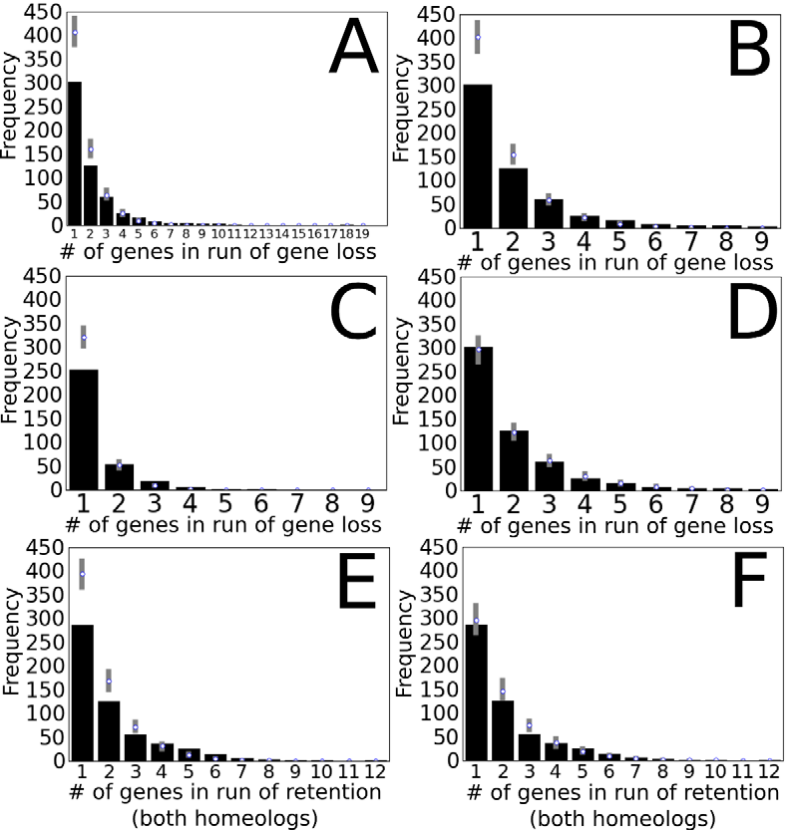
Distribution of runs: Runs of lost genes, or runs of genes retained. *x*-axis: length of the run, in genes. *y*-axis: number of runs. Black bars represent observed data. The white circles within the gray bars represent the median number of runs of that length observed from 1,000 simulations. The gray bars mark the limits within which the values of 95% from the simulations fell. Panels A–D refer to runs of deletions. Panels E and F refer to runs of genes retained from the maize lineage tetraploidy. (A) Observed and distributions of deletion runs in the over-fractionated homeologous regions including all deletion data, and simulated distributions assuming genes are lost solely through a 1 gene deletion mechanism. (B) Observed distributions of deletion runs in the over-fractionated homeologous regions with deletions longer than nine genes removed as likely segmental transpositions, and simulated distributions assuming genes are lost solely through a 1 gene deletion mechanism. (C) Observed and simulated distributions of deletions in the under-fractionated homeologous regions, assuming genes are lost solely through a 1 gene deletion mechanism. (D) Observed and simulated distributions of deletion runs in the over-fractionated homeologous regions, using a model where 80% of deletions are single gene, 15% remove two adjacent genes, and 5% of deletions remove three genes in a row, best fit ratio determined by a genetic algorithm. (E) Observed runs of genes retained in both homeologs and simulated distributions assuming genes are lost solely through a 1 gene deletion mechanism. (F) Observed runs of genes retained in both homeologs compared to the simulated expectation assuming 80% single-gene deletions, 15% two-gene, and 5% three-gene.

We next asked, what was the average extent of gene loss? Most importantly, are deletion events longer than one gene? Figure 4A, B, C, and D monitors runs, or the sequential series of deleted genes, and Figure 4E monitors runs of retained genes. The experimental runs data plotted in Figure 4A, B, C, and E were compared to the 95% confidence interval around the median of Monte Carlo simulated data (Methods) based on the assumption that one gene is deleted at a time, and the chromosome was ligated before the next deletion, as such a mechanism would be predicted to work in nature. The distributions of Figures 4A, B, and C are all very similar: they differ only as to whether or not the over-fractionated or under-fractionated chromosome is evaluated or as to whether or not gene losses of 10 genes or greater were included. The most frequent run length in distributions Figure 4A, B, or C is one gene, followed by two genes, and so forth. If we recalculate expectations for distribution of Figure 4C using an evolutionary method that permits varying percentages of deletions of genes, the best fit is one-gene deletions 80%, two-gene deletions 15%, and three-gene deletions 5% (Figure 4D).

The possibility existed that longer deletion runs were not authentic deletions but were segmental translocations. Deletion runs consisting of 12 genes or more were found somewhere else in the genome. Deletions of between 11 and 6 genes were found elsewhere about 10% of the time, but identification was made more difficult because fractionation is expected to remove genes from any position in the genome, and sometimes is expected to leave behind fewer than the three syntenic genes needed for a positive identification. That is why deletions of 10 or more genes were removed from all distributions of Figure 4 except Figure 4A. There is a possibility, a possibility we evaluate, that the smaller deletions are also undetectable segmental translocations and not authentic deletions, but removal is essentially one gene at a time.

### Clear Differentiation Between Deletion of a Segment of DNA and Translocation of That Segment to a New Position

We next asked if it were possible that, rather than being deleted, single genes observed as lost between orthologous maize and sorghum regions were instead transposed or translocated elsewhere in the genome, as we had observed for longer runs of genes. Large-scale single gene transposition has been documented in the eudicot order Brassicales [9], and cases have also been reported in the maize lineage [11]. To address the possibility that the majority of the fractionating gene loss we were observing was actually a result of whole-gene transposition, we attempted to identify potentially orthologous maize genes in a position-independent manner. Sorghum genes with known orthologs in rice were blasted against sorghum, rice, and maize genomes, as described in Methods. From the resulting data we found that genes identified as retained had a mean of 1.57 copies in the genome, with a median of 2 genes. It is expected that this number would be less than 2, as the manual annotators considered a gene to be retained if a significant fragment of it was still present, which included genes in the process of being removed by small deletions (as will be discussed). Genes identified as being fractionated in Dataset S1 were present at a mean of 1.17 copies in the genome, with a median of 1 gene. A few of these extra copies are likely maize-specific duplications, but others no doubt represent apparently deleted homeologs that have transposed to other locations in the genome. Nevertheless, these data provide strong evidence that while some apparently fractionated genes may have been lost via translocation (transposition to a new site), translocation is not the prevailing mechanism explaining our fractionation data. This conclusion does not imply that fragments of genes are not transposed around the genome, as is known to occur frequently via transposon-mediated gene capture [20]. Indeed, when we re-calibrated our search to find shorter stretches of high-identity sequence, we found many pieces of genes present at higher copy numbers elsewhere in the genome. Examination of a sample of these hits identified gene fragments, but no intact genes were found.

### Genes Retained as Pairs from the Maize Lineage Tetraploidy Are Rarely Clustered Beyond Expectations

As shown in Figure 2, fractionation itself clusters retained genes. Figure 4E identifies runs of retained genes (Bs) and distributes them by run length and compares this to expectations based on deletions one gene at a time. Is this distribution more highly clustered than expected from fractionation alone? The mode is clearly one retained pair, as expected. Expectation intervals were generated assuming that deletions occurred one gene at a time. Although clustering of retained genes is not dramatic, runs of retained genes greater than 9 gene pairs are not expected at all; in total, there are 62 genes (out of 1,203, or 5%) in such longer runs ranging from 9 through 12 genes in length. When expectations are changed to be 80% single-gene deletions, 15% two-gene deletions, and 5% three-gene deletions (Figure 4F), the actual and expected are similar. Now there are only four unexpected runs greater than 9 genes in length. With the exception of these few longer runs, genes are retained approximately as expected based on 80%/15%/5% 1/2/3 gene deletion predictions.

### Use of Whole-Gene Count Data to Evaluate Fractionation Bias Throughout the Genome

Table 1 focuses on nine longer representative homeologous regions of maize representing different sorghum chromosomes. The under-fractionated and over-fractionated homeologs in maize are identified in this table (Column 2). This over/under designation derives from the deletion bias data quantified in Table 1 and evaluated for significance in Column C. Table 2 shows these data for each of the nine representative regions individually (Column H). In this case, the numbers are less than 1 because the ratio is under-fractionated/over-fractionated, and the under-fractionated homeolog has fewer deletions. The homeolog with the fewest deletions contains the most genes, so another measure of fractionation is the number of genes on the under-fractionated/over-fractionated, where bias will now be indicated by ratios greater than 1. The fractionation bias ratios, using total gene data, for each of the nine representative regions are listed in Column L of Table 1.

**Table 2:** Repeat sequence signature analyses of 16 *Zm* deletions within exons of eight genes.

44595 Sorghum Gene Names From Sbi1.4+Version 1 of Freeling Lab Os-Sb CNS Pipeline, 2009 Official Genes Are Given SbXg Numbers by JGI	Gene Description (Based on Oryza Sativa (Os) or ARABIDOPSIS thaliana (At) if Sorghum Description Was Not Available)	*Best Hit in Rice (Oryza Sativa, TIGR v. 5)*	Ungapped Maize Homeolog	Ungapped Maize Chromosome	Gapped Maize Homeolog	Gapped Maize Chromosome	Over-fractionated Maize Chromosome	Repeat Flanking Deletion in Maize Homeolog	gap Size
*Sb01g039030*	putative ankyrin repeat family protein	*Os03g17250*	GRMZM2G042107	1	GRMZM2G020982	9	9	CGAT	12
*Sb01g039030*	putative ankyrin repeat family protein	*Os03g17250*	GRMZM2G042107	1	GRMZM2G020982	9	9	GAAG	12
*Sb01g039030*	putative ankyrin repeat family protein	*Os03g17250*	GRMZM2G042107	1	GRMZM2G020982	9	9	none	14
*Sb01g039030*	putative ankyrin repeat family protein	*Os03g17250*	GRMZM2G042107	1	GRMZM2G020982	9	9	AGG	5
*Sb06g019130*	unknown	*Os04g38920*	GRMZM2G094532	2	RMZM2G107299	10	10	none	8
*Sb06g019400*	putative 18S pre-ribosomal assembly protein	*Os04g39240*	GRMZM2G094532	2	RMZM2G107299	10	10	none	19
*Sb09g023600*	hydrogen-exporting ATPase activity, phosphorylative mechanism	*Os05g40230*	GRMZM2G701207	6	GRMZM2G082721	8	8	CGCCGAGAAGGCCA	55
*Sb09g023840*	putative zinc finger (C3HC4-type RING finger) family protein	*Os05g40980*	GRMZM2G157246	6	GRMZM2G300589	8	8	CCGCCT	9
*Sb09g023840*	putative zinc finger (C3HC4-type RING finger) family protein	*Os05g40980*	GRMZM2G157246	6	GRMZM2G300589	8	8	none	8
*Sb09g023840*	putative zinc finger (C3HC4-type RING finger) family protein	*Os05g40980*	GRMZM2G157246	6	GRMZM2G300589	8	8	CCC	9
*Sb09g023840*	putative zinc finger (C3HC4-type RING finger) family protein	*Os05g40980*	GRMZM2G157246	6	GRMZM2G300589	8	8	none	9
*Sb10g029310*	unknown	*Os06g49180*	GRMZM2G083022	6	GRMZM2G347721 (closest feature)	5	6	CTTAAGAGCGATACCGTGCATCTG	178
*Sb10g030110 (1)*	kinase activity	*Os06g50100*	GRMZM2G158045	5	GRMZM2G146305	6	6	CCCGT	27
*Sb10g030110 (2)*	kinase activity	*Os06g50100*	GRMZM2G158045	5	GRMZM2G146305	6	6	none	12
*Sb10g030110 (2)*	kinase activity	*Os06g50100*	GRMZM2G158045	5	GRMZM2G146305	6	6	GGACT	9
*Sb10g030776*	putative starch branching enzyme	*Os06g51084*	GRMZM2G088753	5	(chr: 6 81555469–81575469)	6	6	GAAAC	27

Note that more than one gap, a single deletion, may exist within the exons identified in our search. In one case, the maize homeolog corresponding to *Sb10g030110* had gaps in two separate exons (denoted as “1” and “2,” respectively).

To extrapolate bias in our manually annotated regions to more of the genome, we used the slope of syntenic lines in *Zm-Zm* dot plots (Dataset S4). A slope of 1 implies unbiased fractionation. A significant difference in the number of genes or base pairs between the two homeologous maize chromosomes alters that slope from 1, and this is what we observed. If the unit of *Zm*-under/*Zm*-over measurement is total number of genes annotated by maizegnome.org, the average slope value corresponds to a mean fractionation bias value of 1.5 (Table 1, Column M). If the units are in total base-pairs, the fractionation bias is 2.3 (Table 1, Column N). Again, the under/over direction of fractionation in both cases remains greater than 1, as expected, but the dot-plot analysis made it possible to examine considerably longer regions of paired homeologs, each anchored on the indicated sorghum region. Most important here is that the three measures of bias based on gene number (Columns L and M) or base pair length (N) are concordant with expectations based on the rigorous deletion bias data generated manually for our representative regions. Based on the concordance between bias in orthologous gene loss and base pair length, and given that 85% of the maize genome is composed of transposable elements [13], we conclude that homeologous regions that preferentially lose genes also lose intergenic, primarily transposon, DNA more frequently.

### The Under- and Over-Fractionated Maize Homeologs Are Equally Diverged from Their Sorghum Ortholog at the Level of Nearly Neutral Base Pair Substitution (Ks)

Table 1, Column O, reports our measured ratio of Ks [*Zm*-under/*Sb*] to Ks [Zm-over/*Sb*] for a total of 1,772 *Sb-Zm-Zm* gene units in the five sorghum homeologous regions for which highly significant under/over-fractionation expectations existed (Table 1). We removed 16% of pairs with the most extreme Ks ratios, many of which represent misalignments or alignments to pseudogenes. Using the remaining data, we found no difference between the Ks values between sorghum and either of the two maize homeologous regions. We conclude that mutation by base substitution and mutation by short deletion are mechanically distinct and are targeted differently.

### There Is No Obvious Correlation between an Over-Fractionated Chromosomal Arm and the Number of Map Units (% Recombination) in That Arm

Three of our representative regions are within pairs of homeologous chromosomal arms: *Sb1* = *Zm*1S/9L (sorghum chromosome 1 = maize chromosome 1S and maize chromosome 9L), *Sb3* = *Zm*3L/8L, and *Sb*6 = *Zm*2S/10L. The under-fractionated (longer) homeolog is the numerator. These are the only arm-arm exact homeologies in the maize genome; examination of syntenic *Sb-Zm* dot plots (like that in Dataset S2) made clear that segments of these arms are not present syntenically on any other chromosomes. The total map unit's length of these maize chromosomal arms is known, making it possible to directly compare the degree of fractionation within any given arm to the overall recombination frequency within that arm. Mapping data for maize inbred T232×x inbred CM37 generated the following data [21]: the proportion of map units for under-fractionated arm/over-fractionated arms are *Zm1S/9L* = 0.9, *Zm*3L/8L = 1.1, and *Zm*2S/10L = 1.9 (Table 1, Column N). Note that although *Zm*2S/10L has the largest difference in recombination frequency, it has the lowest fractionation bias of these three paired arms (Table 1, Column C). We conclude that there is no obvious correlation between biased fractionation and overall frequencies of reciprocal recombination during meiosis.

### Quantitative Estimate of Methylation Status for Over- and Under-Fractionated Maize Homeologous Chromosomal Segments

Even before BAC sequencing was complete, one group [22] identified methylation domains of maize chromosome in shoot or root nuclei using McrBC restriction endonuclease, a treatment that degrades DNA between methylated half sites of the form m5C-N40–500-m5C. McrBC is non-specific for different types of methylation patterns. Using this crude measure of methylated regions (BAC start-stop) in maize shoot nuclei, we overlifted (translated the start-stop nucleotide designations) the data from BACs to pseudomolecules and found no correlation at all between the over-fractionated and under-fractionated homeologs (Table 1, Column M). Two representative regions were concordant, two were not concordant, and one region was vastly over-methylated on the under-fractionated homeolog.

### Single Deletions within Exons Occur Primarily on the Over-Fractionated Chromosomes and Appear to Be Due to Illegitimate Recombination

**Figure 5:**
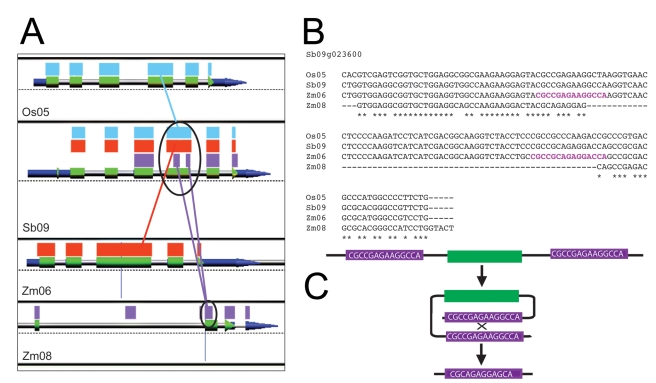
Deletions and intra-chromosomal recombination in fractionated maize homeologs. (A) A cartoon of a blastn output between orthologous genes in rice, sorghum, and two maize homeologs. The colored rectangles (blue, orange, purple) represent high-scoring sequence pairs (HSPs), or regions with high sequence similarity to each other. Sorghum is the reference sequence (Sb09). The top two panels show the syntenous exon sequence (highlighted in blue) between rice (rice chromosome 5, or Os05) and sorghum (sorghum chromosome 9, or Sb09); the second and fourth panels demonstrate the location of the deletion in maize homeolog *Zm* chromosome 8 (Zm08) to its sorghum ortholog (purple). As can be seen, the deletion is evident when the Zm08 sequence (circled) is compared to the orthologous sequence of Zm06 (orange) and rice (blue) when all HSPs are aligned on sorghum. The GEvo alignment output for these data may be found at http://genomevolution.org/r/3em. (B) A ClustalW alignment of the rice, sorghum, and maize homeologs from Figure 5A. The purple sequence in the unfractionated maize homeolog (Zm06) indicates the location of the direct repeat sequence that originally flanked the deletion in the fractionated homeolog (Zm08). (C) A diagram representing the mechanism of intra-chromosomal recombination, based on the flanking sequence highlighted in (B). Direct repeats come together to form a circle, which is then recombined away, leaving a solo repeat in its place.

Our methods for deciding whether or not a maize gene was retained (“B”) did not require that the entire coding sequence be present, but only a significant fragment. Because of this, our calculation of the number of whole sorghum-maize genes retained post-tetraploidy, about 40%, is surely an overestimate. If we were to assume that the process of fractionation is ongoing, we reasoned that some of our retained genes might have internal deletions whose flanking sequences might give us a clue as to the mechanism behind gene fractionation. By visual examination, we identified cases where a maize gene seemed to have a gap within an exon. To verify each fully flanked deletion, we extracted the sorghum exon sequence and used it as query for a blastn to rice, a grass that diverged from sorghum about 50 MYA [23], to sorghum itself, and to the two homeologous maize regions. We then studied each *Os-Sb-Zm1-Zm2* blastn result using GEvo, our synteny visualization platform (in CoGe, Methods). We verified that eight genes, containing a total of 16 deletions, were fully flanked by conserved, known sequence. Table 2 gives the data for these fully flanked deletions. Figure 5A and B shows an exemplary GEvo graphic and the pertinent orthologous sequences of rice, sorghum, and the two maize homeologs. In two cases (*Sb01g039030* and *Sb09g023840*, Table 2), the apparent gap was actually several short gaps within the homeologous flanking sequence. The gap size within these 16 deleted regions ranged from 5 bp to 178 bp, with a mean gap size of 25.9 bp. Bias for gaps is consistent with the fractionation bias found locally: in other words, when a gap is present, it is in the maize homeologous gene located on over-fractionated chromosome 93% (15/16) of the time (Table 2).

As mentioned in the Introduction, deletions due to illegitimate recombination are often flanked by a short stretch of sequence that, before the deletion, had been a direct repeat [11]. In theory, such repeats facilitate ectopic, intra-chromosomal, reciprocal recombination (as drawn in Figure 5C) generating a circle and a solo copy of the original repeat sequence in place of the sequence deleted (the circle). Using ClustalW, we found such direct repeats flanking 10 of the 16 gaps in our study (Table 2). These repeats were between 3 and 24 bp in length; an example is given in Figure 5B. Notice how the repeats surrounding the gap in the fractionated homeolog are truncated in comparison to the repeat sequence within the whole homeolog: this is a typical footprint of intra-chromosomal recombination [11].

In an attempt to generalize our results from monocots (e.g. grasses) to eudicots (e.g. legumes), we found several such small deletions where the inferred precursor sequence was flanked by direct repeats, within retained duplicate genes of *Glycine max* (*Gm*: soybean, unpublished data) from the more recent of the two easily observable tetraploid events in the sorghum genome. Soybean has had two recent genome duplication events, the most recent one (alpha) having taken place between 14 and 3 MYA [14]. The close relative, *Medicago trunculata*, was used as the outgroup in order to infer the precursor gene sequence before deletion. We conclude that small deletions are involved in the fractionation of genes following ancient (successful) plant tetraploidies.

## Discussion

Comparison of the sorghum outgroup to the newly released maize sequence permitted a detailed description of the consequences of tetraploidy and the ensuing fractionation process on grass genes shared orthologously between sorghum and maize. We used graphic displays of blast results, both as pairwise dot-plots (SynMap) and multiple ortholog line drawings (GEvo), to facilitate large-scale genome analyses at the level where 100 bp deletions from genes were observed visually. The maize tetraploidy is much more recent than the previously studied alpha tetraploidy of Arabidopsis. Combining the power of the sorghum outgroup and the recent and potentially ongoing fractionation of the maize genome permitted a definitive description of the sequences left after fractionation. We observed: (1) If we define a gene stringently, then it appears that fractionation generally involves gene deletion, not gene repositioning. However, if we define a gene as a 150 bp fragment of exon, significantly more transposition/duplication is evident. Any transposon-capture [20] or fragment transposition mechanism could help explain these results. (2) If the unit of deletion is “genes,” then the deletion mechanism of fractionation most frequently removes one gene (Figure 4). Indeed, our best-fit evolutionary model for predicting the actual gene loss on the over-fractionated chromosome was the loss of one gene 80% of the time, the loss of two genes 15% of the time, and the loss of three genes 5% of the time. The genes that resist fractionation are naturally clustered by fractionation, as predicted, though a few runs of retained genes are unexpectedly long (Figure 4E and F). (3) The lower limit of gene loss was estimated from those infrequent deletions that were completely contained within an exon; these ranged from 5–178 bp in length. We think it likely that these intra-exon deletions are the consequence of a single event rather than the summation of an ongoing series of events. Because single genes were found with deletions in more than one exon, it is clear that smaller deletions (less than 200 bp) are common, but larger deletions also sometimes happen. We also found evidence that illegitimate recombination acts in soybean as it does in maize (unpublished data), so this mechanism is not maize-specific. (4) By adding the orthologous rice genes to the *Sb-Zm-Zm* panel, we inferred the sequence of the maize ancestral chromosome before the small deletions described above took place. The ancestral to-be-deleted sequence was flanked by a direct repeat of between 3 and 24 bp in length. Such flanking repeats have been interpreted as signatures of illegitimate recombination. One such mechanism is intra-chromosomal recombination, which pairs on the direct repeat and generates a circle and a deletion [12]. (5) Overall, one homeolog is, on average, 2.3 times more likely to have a gene removed by deletion than the other homeolog, demonstrating biased fractionation. Biased fractionation was also seen by the team of researchers who collaborated to first describe the maize genome [13]. That the DNA between genes on the over-fractionated chromosome are even more over-fractionated than the genes themselves—DNA composed primarily or entirely of transposons thought to be without function—makes it unlikely that fractionation bias is the result of any sort of selection bias. (6) We found no correlation between *Sb-Zm* Ks values with over/under-fractionation. Divergence by point mutation and fractionation by short deletion are independent and independently regulated. (7) Preliminary identification of methylation domains in maize [22] permitted an attempt to correlate the number of such domains with over- or under-fractionation. We found no such correlation, but this does not rule out other types of epigenetic marks (e.g. histone modification) as possible tags for biased fractionation. (8) Although we implicate some sort of recombination mechanism to facilitate short deletion, there is no correlation between maize chromosome arms that are over/under-fractionated and the number of total map units (% reciprocal recombination) in those arms.

Our detailed analysis evaluates one outcome of the maize alpha tetraploid fractionation, based on the B73 inbred line. Since gene fragments remain, we have no reason to believe that fractionation is complete, and if not complete, then it is probable that different accessions of the species *Zea mays*, and perhaps different inbred lines of the *Zea mays mays* subspecies, have different fractionation outcomes.

We do not know how many individual deletions, on average, it takes to completely remove a gene. However, the observation that 93% of the deletions we found within exons were on the over-fractionated homeolog probably reflects the general scenario: one of the two homeologs is inactivated by deletion, at which point deletions of the other homeolog are selected against (since this second deletion would result in the loss of the function encoded by the gene pair). Additional deletions would then accumulate only on the homeologous gene that suffered the original loss as fractionation of this now-inactivated gene progressed. Even so, it took little effort to find a case in soybean where a flanking repeat signature implied that an entire gene was removed in one deletion event (Dataset S5) from a region where there were few exon deletions. We do not know unequivocally the relative frequency of this sort of larger deletion compared to genes being deleted away in smaller increments. Perhaps the nature and distribution of direct repeats, the length of the circle to be deleted, and the epigenetic receptivity of the target chromosome all contribute to the details of fractionation.

Sometimes genes that resist fractionation, the retained genes, are significantly clustered ([24] and Figure 2) beyond expectations derived from any mechanism of gene deletion. One explanation for this could be that genes that would be otherwise fractionated are protected by their position next to a fractionation-resistant gene. Alternatively, fractionation-resistant genes might exist as clusters in the pre-tetraploid ancestor.

There are two occurrences of particularly large genomic consequence that happened along the maize lineage only after the divergence of maize and sorghum. First was the maize alpha tetraploidy event that is thought to have occurred roughly 12 MYA. Second, and later, was a massive bloom of transposable element activity, resulting in a modern maize genome 3.4 times as large as that of sorghum. About 85% of maize's 2,300 Mb genome is thought to be composed of transposons [13], many of which inserted within the last 3 million years [25]. Illegitimate recombination has been proposed as a mechanism for genome-size reduction—transposon removal—independently in maize and Drosophila [11],[12]. On a similar theme, some indels within genes in Arabidopsis appear to be due to illegitimate recombination [26]. Our evidence for ancestral flanking direct repeats, and our evocation of intra-chromosomal recombination, are therefore consistent with these previous studies. Unlike previous work, we have focused on typical genes that are targets of fractionation in order to address the mechanism of gene loss following tetraploidy. We now propose that illegitimate recombination is the primary means by which excess DNA in the form of redundant genes and transposons are removed from genomes. Intra-chromosomal recombination is one way to envision this sort of recombination, but any chromosomal complex that deletes between tandem repeat sequences would fit our data. This mechanism is a check against what has been called a “one-way ticket to genomic obesity” [11]. That is not to say that this mechanism evolved in any sort of purpose-oriented (teleological) way.

The sort of purifying selection via deletion we observe in maize is very different from that described for primates, where genes are removed via the pseudogene pathway. For instance, the components of a pheromone signal transduction pathway lost in old-world monkeys, including humans, are still present in the form of identifiable pseudogenes [27], and recent work indicates that 100% of human-specific gene losses among the primates studied are present in the genome as pseudogenes without deletions [8]. It is possible that mammals and plants evolved different mechanisms for genome purification, adapted to fit differences in their capacities to cope with high frequencies of individuals carrying DNA deletions without going extinct.

Unlike transposons, coding regions, such as exons, do not have built-in long direct repeats and do not present obvious targets for illegitimate recombination. Nevertheless they do have randomly situated, shorter direct repeats, and we now know that some of these short repeats facilitate small deletions. An accumulation of such deletions could eventually lead to the disappearance of entire genes. Additionally, deletions in the cis-acting regulatory regions near genes could hypothetically give rise to a new regulatory binding activity. The same can be said for cis-acting regulatory sequences that affect a local chromosomal region rather than a single gene. Following deletion of intervening genes on fractionated chromosomes, new clusters of genes would be expected to respond in new ways to their local regulatory environment. Thus, in large and small ways, the fractionation mechanism we describe has the potential to create huge regulatory variation around genes as a by-product (or “spandrel”) of purifying selection. Whether or not the fractionation mechanism is induced by “excess” is not yet known.

This discussion is not complete without considering the origin and utility of fractionation bias itself. The alpha-syntenic genome of maize is actually two genomes, the over-fractionated and the under-fractionated, and the total DNA and gene count differences between them are diagnostic for any longer stretch of chromosome. We show that Ks data neither support nor refute allotetraploidy. Allotetraploidy—for example, a tetraploidy following a very wide cross—could explain the origin of over- and under-fractionated genomes, where one of the genomes acquired an “invader” epigenetic tag in the new polyploid. Alternatively, the tetraploidy might have been autotetraploidy, and the mode of sexual transmission generated a genome-wide epigenetic tag. Either way, logic alone dictates that some sort of heritable genomic mark precedes the bias in fractionation since biased fractionation is ongoing. One immediate benefit of having such a tag could be to prevent homeologous pairing and consequent dysfunctional pollen and eggs. We do not have any direct data at the level of DNA or histone modification. We also do not know anything about the relationship between chromosome pairing/mispairing and the inferred epigenetic mark.

In summary, we suggest that direct repeats throughout the genome facilitate frequent and continuous sequence deletion via illegitimate recombination. Repeats abound, so targets are not limiting. Among the evolutionary benefits of this selectively neutral deletion/fractionation mechanism is bulk DNA removal and the wholesale generation of new combinations of regulatory and coding sequences. Both tetraploidy and transposon blooms confront the genome with a great deal of potentially dispensable DNA, and both cases of genomic excess probably share the same purification mechanism: intra-chromosomal recombination. Fractionation bias demonstrates that the frequency of this mechanism can be modulated. The inducibility, target specificity, and rate modulation of purifying selection via illegitimate chromosomal recombination is a particularly important subject for further research.

## Methods

### Plant Genomic Sequences

The sorghum sequence was Sbi1 assembly and Sbi1.4 annotation (Paterson et al. 2009 [23]) downloaded from Phytosome V4.0 http://www.phytozome.net/sorghum, last modified 3-25-08. The B73 maize genome sequence was obtained in the form of pseudomolecules in 3-09 (ftp://ftp.genome.arizona.edu/pub/fpc/maize/) and stored in our CoGe platform as database 8082: http://synteny.cnr.berkeley.edu/CoGe/OrganismView.pl?dsgid=8082; and with draft models annotations in 10-09 from maizegenome.org (http://ftp.maizesequence.org/release-4a.53/). The sequence of these two releases is identical. The draft annotated maize sequence will be called “4a.53” in the few instances where we use the official CDS models. The TIGR 5 *Nipponbare* rice assembly and annotation was downloaded onto our CoGe platform (http://synteny.cnr.berkeley.edu/CoGe/) before the MSU6 update file://localhost/ (http://rice.plantbiology.msu.edu/data_download.shtml), and was used in 2008 to generate the sorghum gene list used here; the differences between rice TIGR5 and MSU6 annotations are of no consequence to this project. The soybean and *Medicago trunculata* genomes were downloaded from Phytosome 4.0 http://www.phytozome.net/soybean.php and http://www.phytozome.net/medicago.php, respectively, in early 2009.

### The Sorghum Gene List (Dataset S1)

Sorghum genes were Sbi1.4 to which we added many genes on the basis of orthology to rice *Nipponbare*, TIGR 5; the added genes included many with corresponding RNAs since these are absent in Sbi1.4. Dataset S1 uses the format *Sbxgxxxxxx* for Sbi1.4 genes and *sorghum_chrmosomex_startx_stopx* for genes we added based on *Sb-Os* orthology. The detailed syntenic alignment of the entire genomes of sorghum and rice was automated and frozen in September 2009 as the rice-sorghum CNS discovery Pipeline 1.0. Dataset S6 diagrams this pipeline and details each step. What is most important is that any sorghum gene we use in this analysis is shared syntenically between sorghum and least one of the two possible homeologous maize positions. Some of our added sorghum genes are shared with maize as orthologs; those that are not shared with maize were not studied.

After adding 10,585 new putative genes to the 34,003 official JGI (Joint Genome Institute) sorghum genes, the augmented sorghum genome was masked for any sequence repeated over 50 times, and then everything but exons or RNA-encoding sequence was additionally masked. This heavily masked sorghum genome was then used to query the maize genome.

We found a total of 37 orthologous regions between sorghum and the corresponding maize homeologs retained after the maize alpha tetraploidy event (*Sb-Zm1-Zm2*) in two ways, and both ways used applications available online in the CoGe comparative genomics platform (http://synteny.cnr.berkeley.edu/CoGe/). Central to our success was our ability to clearly visualize the locations of the many translocations and inversions that happened in both the sorghum and maize lineages. Knowing all breakpoints makes it clear that any single sorghum chromosome is orthologous to exactly two maize chromosome regions, even though many smaller segments are often involved (Dataset S2). To this end all of the 37 regions begin and end with at least one gene retained by both maize homeologs. In this way (Methods), a total of 4,461 sorghum genes (10% of the sorghum genome) were set up for manual evaluation. In order to define those genes that had an ortholog in maize, we condensed all members of locally duplicated arrays into one gene and discarded those 492 duplicates (11%), leaving one parent gene for each array. We also invalidated 74 genes that had annotation incongruencies, and then disregarded another 953 genes for which we failed to find maize orthologs. Each sorghum gene is given an evaluation code of “1” (has an ortholog in the first *Zm* homeologous region), “2” (has an ortholog in the second *Zm* homeologous region), or “B” (has an ortholog in both *Zm* homeologous regions). The designations “O,” “N,” and “D”: D = local duplicate; N = invalid data; O = no ortholog in *Zm*. In each of the 37 regions within Dataset S1, every sorghum gene has been annotated with one of these six symbols. A link (tinyurl.com, a URL abbreviation service, or genomevolution.org) is provided for each *Sb-Zm1-Zm2* panel to facilitate the repetition of our research in the GEvo alignment graphic tool we used for research (Dataset S1). We were left with 2,943 orthologous *Sb-Zm1-Zm2* genes spread over 37 *Sb-Zm1-Zm2* regions.

### Using Applications in the CoGe Toolbox, Including the Ks Values Provided in SynMap

CoGe is an integrated collection of maintained databases, algorithms and applications useful to compare complete genomes on demand [28],[29] and without which it would be difficult to perform our analyses in a reasonable amount of time. SynMap, within CoGe, is a dot plot application that implements the DAGchainer algorithm [30] to identify syntenic lines in two-dimensional arrays of blastn hits between two identical (to find homeologies) or different (to find orthologies) genomes. Each “dot” is a gene pair. The color of this dot can be portrayed to reflect Ks (synonymous base-pair substitution frequency), so syntenic lines of different ages have different colors (see Dataset S2). Clicking on any dot in SynMap anchors the GEvo sequence comparison tool and automatically generates a blastn alignment output. Each output (like a BLAST or LAGAN output) includes a graph, a link, and can be repeated on demand with different settings. CoGeBlast takes sequence from any other CoGe applications or text as query to any number of genomes; the blastn or tblastx results may be downloaded into GEvo panels. GEvo panels may be combined via links to create experiments.

Ks values may be calculated for each data point in SynMap if the genomes being compared are repeat-masked and have annotated CDS sequences. The 4a.53 maize sequence was used. Several genomic comparisons in SynMap have Ks values pre-calculated, including sorghum/maize. Syntenic gene pairs were identified by using blastn with SynMaps's default settings [−W (word size) = 11, −G (gap open) = −1, −E (gap extend) = −1, −q (mismatch) = −3, −r (match) = 1] and an e-value cutoff 0.05. These pairs were used to identify any putative homeologs between coding sequences using DAGchainer to identify collinear sets of putative genes with the following parameters: −D = 20, −g = 10, −A = 5. Ks values for syntenic gene pairs were calculated by first performing a global alignment of virtual protein sequences using the Needleman-Wunsch algorithm [31] implemented in python (http://python.org/pypi/nwalign/). The BLOSOM62 scoring matrix was used for the alignments [32]. From these protein alignments, the codon DNA alignment was generated through back-translation. Ks values were calculated using codeml of the PAML software package [33] on the codon alignment with the following parameters: outfile = mlc, aaDist = 0, verbose = 0, noisy = 0, RateAncestor = 1, kappa = 2, model = 0, ndata = 1, aaRatefile = wag.dat, Small_Diff = .5e-6, CodonFreq = 2, runmode = −2, alpha = 0, omega = 0.4, fix_kappa = 0, Mgene = 0, method = 0, fix_omega = 0, getSE = 0, NSsites = 0, seqtype = 1, cleandata = 0, icode = 0, fix_alpha = 1, clock = 0, ncatG = 1, Malpha = 0, fix_blength = 0. This pipeline is part of the SynMap application in the CoGe suite of comparative genomics software, and its dotplot visualization tool was used to generate the Ks color-coded lines of Dataset S2, and its text output was used to supply the Ks values for the “sorghum/maize Ks differences” Methods section to follow. Since this Ks pipeline will calculate Ks values for erroneously aligned pairs, values far off from an expected normal distribution for any experiment were discarded.

### Preparing the *Sb-Zm1-Zm2* Sequences for Comparison

The entire sorghum genome was subjected to a 50× repeat mask, where every base pair that was covered more than 50 times by a blast hit from a whole-genome self-self blast was masked, using parameters of blastn at word size 16 and e-value cutoff of 0.001. Repeats over 50× genome-wide were masked by changing their sequence to “x.” We needed to use a step-wise approach to accomplish the same 50× mask for maize because a direct self-blast was too memory-intensive for our computers. First we self-blasted pseudomolecules 1–3 as if they were the whole maize genome and masked their 50× repeats. Then we added these 3 larger masked chromosomes to the other 7 unmasked and performed self-BLAST—as with sorghum above. Repeated sequences are color-coded pink in Figure 3, panels B and C. The sorghum 50× masked genome was further masked for every sequence that is not either an Sbi1.4 exon or other sequence shared orthologously with rice, as derived from the rice/sorghum Pipeline 1.0. The non-exon, non-conserved sorghum sequences masked by this method are colored orange in our GEvo graphics (e.g. Figure 3, panel A).

The nine *Sb-Zm1-Zm2* regions were derived from SynMap blastn [34] dotplots using the DAGchainer settings −g = 10 genes, −D = 20 genes, −A = 5 genes, and a Ks color code that clearly distinguishes syntenic lines reflecting sorghum/maize orthologs to lines reflecting more ancient syntenies. When a single stretch of sorghum clearly hits two longer stretches of maize, the center of the overlapped region was used as an anchor to create *Sb-Zm1* and *Sb-Zm2* GEvo panels, which are then combined into a single view. The sorghum/maize Ks-colorized dotplot can be seen in Dataset S2, where the identification of *Sb1* is illustrated. It is possible to regenerate a near-identical graphic in CoGe by visiting http://tinyurl.com/ygx2apu. The 28 additional Sb*-Zm1-Zm2* regions were discovered by choosing as query exons from sorghum genes that encode transcription factors. Each query found, using CoGeBlast, two orthologs in maize about one-third of the time. From CoGeBlast output, it is easy to create *Sb-Zm1-Zm2* GEvo panels. Lengths of these three chromosomes were adjusted so that a chosen segment of sorghum begins and ends with a retained gene, was entirely represented syntenically within the two maize segments, and syntenic coverage did not improve by adding 500 kb on both sides of the maize chromosomes. Inversions do not cloud our analyses because all inversions we include begin and end within each region.

Our primary data of Dataset S1 required that every gene on the sorghum gene list receive one among several possible annotations. Genes in local arrays were marked as parent, duplicate (D or DUP), or interrupter (a gene located within a tandem repeat) using published methods [7] and duplicates were marked and ignored subsequently; up to three interrupter genes were permitted. If a remaining gene occurred syntenically (blastn bitscore >50) on a maize homeolog, then it was coded “1” or “2” if it occurred on only one of the homeologs or “B” if it occurred on both. A few genes were invalidated for technical reasons, and some genes were not found in the syntenic position in either maize homeolog (encoded as “0”). Genes represented by fragments were counted as “present” even though they were almost certainly in the process of removal. In this manner, each of our 37 *Sb-Zm1-Zm2* regions were reduced to a code of *shared genes*, like B1122BBB12BB2B21BBB11B21B, and trimmed to begin and end with a B (present in both maize homeologs) where the terminal Bs were not within inversions. For the diagrams of Figure 3 B, C, and D and Dataset S2, and for all analyses of runs, as discussed in the text we removed runs of 1's or 2's that extended beyond nine genes. This is because our analyses suggested that a run of 10 or more 1's indicates that the 10 genes that would be the corresponding 2's had jumped elsewhere in the genome. The unmodified data are in Dataset S1. At this time, accurate fractionation annotations would be difficult or impossible to achieve automatically largely because of biological complications involving inversions and also by contig misalignments during sequence assembly.

### Fractionation Statistics

The binomial test was used to evaluate the probability that the ratio of deletions on the maize homeologs could occur by chance given an expectation that a single deletion is equally likely to occur on either homeolog. The distribution of all observed deletion lengths is plotted in Figure 4 as the blue bars for the over- and under-fractionated homeologs. Using the initial hypothesis of a deletion mechanism that independently eliminates one gene at a time, a simulation of gene loss was carried out. Starting with a length equal to all genes, both deleted and still present, genes were deleted at random until the simulated number of deletions was equal to the true observed number. The distribution of apparent deletion lengths for the run was then saved and the preceding steps were repeated 1,000 times. This gives a distribution of frequencies of all deletion lengths. The median number of apparent deletion runs from these simulations is shown by the white circles in the grey lines of Figure 5, with grey line itself marking the values between which the results from 95% of the simulations fall.

For Figure 5E, which plots runs of genes conserved on both maize homeologs, the above model was modified by generating two lengths each equal to the total number of sorghum genes within the dataset, and then deleting genes from either one or the other sequence (with an bias for deleting genes from one or the other dataset equal to that observed in the overall fractionation dataset) until the number of retrain genes (Bs) was equal to the true number observed, with the constraint that once a simulated gene was deleted from one dataset, the orthologous gene in the other dataset would never be deleted.

As the simulated distribution did not perfectly match the observed results, a genetic algorithm using 20 (genetic) character states, each representing a 5% (1/20) chance that a deletion would be some length between 1 and 5 genes long was used to determine, given the region length and the distribution of observed deletion lengths, the ratio between different deletion lengths to use in the simulation described above to achieve the best match between simulated and observed data. The fitness of solutions in the evolutionary algorithm were scored using the Monte Carlo method described in the proceeding paragraph (with the modification that rather than fixing the deletion length at 1 gene, deletion lengths were selected using the weighted averages generated by the evolutionary algorithm) with the most fit solutions being those where the median simulated number of deletion runs was least different from the observed number of runs. The genetic algorithm was allowed to run for 100,000 generations. These new weighted average deletion lengths can then be used to generate new sets of expectations for data, as seen in Figure 4D. The script used to run the genetic algorithm is available at http://code.google.com/p/bpbio/source/browse/trunk/scripts/fractionation/fractionation_ga.py and in Dataset S7.

### Finding Segmental Translocations

Sorghum genes with known orthologs in rice were blasted against the sorghum (JGI 1.4 gene models), rice (TIGR 6.0), and maize (4a.53 filtered gene set; maizegenome.org) datasets. We used the score of the best sorghum-rice alignment as a cut-off to avoid hits from genes that diverged before the rice sorghum split, and removed genes with more than one hit above that threshold in the sorghum-sorghum blast to avoid the inclusion of genes that duplicated in the sorghum-maize lineage since the divergence from rice. These criteria left us with a set of approximately 10,000 genes with a single hit in sorghum that had a greater bit score than any hit in rice, and one or more hits satisfying the same conditions in maize. 406 genes from this dataset overlapped with genes identified as retained (noted as “B” in Dataset S1) by manual annotators, and 771 overlapped with genes identified as fractionated. Stretches of 10 or more genes deleted from the same chromosome were identified on Dataset S1 and the missing region was identified by a discontinuity in the appropriate sorghum/maize dot plot. We built a string of exons that identified each gene in the deleted region and used it as query to the subject maize genome. The maize genome was 50× repeat-masked, as described, and blastn used settings of word size 7, and e-value <0.001. Hits were achieved in CoGeBlast and evaluated in GEvo. Any three of the expected genes, arranged syntenically, in unexpected regions of genome were taken as evidence for a segmental translocation even though a gene might have been represented by a fragment rather than an entire gene.

### Evaluation of Copy Number of Genes in the Nuclear Genomes of Sorghum and Maize

The coding sequences of the subset of genes from the JGI sorghum 1.4 gene set that had been identified as orthologous to a single rice gene were blasted against the MSU6 rice gene set and the maize 4.a53 filtered gene sets as well as against the same sorghum gene set. For each sorghum gene, the bit score of the highest-scoring alignment against a rice gene was used as a cutoff to exclude hits from genes that had diverged from the gene being tested before the rice/sorghum split. Sorghum genes that hit one or more additional sorghum genes with bit scores higher than that cutoff were excluded from the analysis to exclude genes duplicated in the maize/sorghum lineage since the rice/sorghum split.

The number of hits to genes in the maize filtered gene set for the remaining sorghum genes (with scores higher than the best hit in rice) was recorded. After the accuracy of a sample of the results were manually checked using CoGe, the final data were generated by looking at the average number of maize genes found using this process for genes assigned to the fractionated and unfractionated categories by manual annotation.

### Looking for *Sb-Zm* Ks Differences Depending on Whether Over- or Under-Fractionated

Ks values for shared open reading frames in sorghum and maize (4.a53) were precalculated and loaded into SynMap, in CoGe as described previously in METHODS. The sorghum/maize orthologs that also fell into the 37 regions that were hand-annotated for the primary fractionation data (Dataset S1) were identified. Next, sorghum genes that hit to genes in both maize homeologs (encoded “B”) were paired and their Ks values compared. Data were reported in the format *Sb-Zm1*-under-fractionated/*Sb-Zm2*-over-fractionated. Visual examination of the Ks data showed a minority portion of very extreme ratios, likely the result of misalignments, alignments to pseudogenes, or alignments to non-orthologous genes. Such misalignments were expected due to the fragmented nature of many B73 genes and contig assembly error. The 16% of pairs with the most extreme ratios as compared to the median were removed from the dataset and not used to calculate results.

### Counting Methylation Domains on Maize Chromosomes

We overlaid McrBC methylation data from [22] onto the annotated maize pseudomolecule sequence (dataset *Zm* 4a.53) and uploaded the modified database into the genome viewer we use with CoGe: GenomeView. We were able to visualize on GenomeView the locations of methylated sites on maize chromosome regions. After anchoring both maize homeologs to their orthologous sorghum sequence with the stop-start sites used in our fractionation analyses, we manually counted the number of methylation peaks in each maize homeologous region in question.

### Analyses of Deletions within Exons

Using GEvo, with parameters set for blastn with a spike-length of 15 bp, we visually scanned all retained maize genes from our *Sb/Zm1/Zm2* dataset to look for gaps within exons of one or the other maize homeolog. This level of resolution did not permit us to identify single gaps less than approximately 15 bp long. However, we did not intend to be exhaustive. Once a gap was identified, we extracted the sorghum exon sequence and used it as query in a blastn comparison to rice; this use of the rice as a secondary outgroup often confirmed the sorghum full-length exon annotation, and when it did, we re-blastn'd this sequence against the multiple subjects rice (*Oryza sativa* v5 masked repeats 50×X), sorghum (vSbi1.4 exons, 50×X mask+syntenic thread with *Os*), and maize v4a.53 to produce GEvo images like that shown in Figure 5A. We then took the corresponding exon sequence data from rice, sorghum, and both maize homeologs and used ClustalW (http://www.ch.embnet.org/software/ClustalW.html) to visualize the sequence alignment surrounding the gap, as well as the sequence on the homeolog without the deletion (as in Figure 5B).

## Supporting Information

Dataset S1 **The sorghum gene list.** Sorghum genes from 37 regions were from Sbi1.4 to which we added many genes on the basis of orthology to rice *Niponbarre*, TIGR 5; the added genes included many with corresponding RNAs since these are absent in Sbi1.4. SI1 uses the format *Sbxgxxxxxx* for Sbi1.4 genes and *sorghum_chrmosomex_startx_stopx* for genes we added based on *Sb-Os* orthology. Genes in local arrays were marked as parent, duplicate (D or DUP), or interrupter (a gene located within a tandem repeat) using published methods [7], and duplicates were marked and ignored subsequently; up to three interrupter genes were permitted. If a remaining gene occurred syntenically (blastn bitscore >50) on a maize homeolog, then it was coded “1” or “2” if it occurred on only one of the homeologs or “B” if it occurred on both. A few genes were invalidated for technical reasons (“N”), and some genes were not found in the syntenic position in either maize homeolog (encoded as “0”).
(2.37 MB DOC)

Dataset S2 **The sorghum-maize dot-plot.** Sorghum (*x*-axis) and maize (*y*-axis) with alpha-tetraploidy lines colored purple by lower Ks from SynMap in CoGe. Numerals are chromosome numbers. Lower Ks is more recent. Although hundreds of breakpoints are evident, each segment of maize is orthologous to one sorghum region, and each sorghum segment is orthologous to two maize regions.
(0.38 MB PDF)

Dataset S3 **Fractionation runs used to determine bias for all 37 orthologous sorghum/maize regions.** Here, bias is measured in units “genes lost completely.” The code we used, taken from the Dataset S1 datasheet (e.g. 11BBB1121B2121BBBB2222BB…), is given at the top of each diagram. Assuming that genes are lost in units of one gene, the null hypothesis is that the same number of genes are lost on each of the homeologs: using the symbols of the alignment diagrams, 0 = 1. The *p* value predicts the chance that this 1∶1 ratio is possible. Many genes coded “B” (retained) were actually a complete gene paired with a gene fragment, as expected if fractionation is not complete. All of our 37 diagrams had runs of over nine genes removed because they are known to be segmental translocations.
(0.04 MB PDF)

Dataset S4 **Maize-maize self-blastn dot-plot.** Sequences present 40×X in the genome were masked. Axes are in genes from annotated psudomolecules from 10-09. Tangent angles = bias. Green lines are higher Ks and are from the alpha-tetraploidy.
(1.60 MB PDF)

Dataset S5 **Whole-gene deletion in soybean (** ***Glycine max*** **).** (A) A GEvo output of soybean homeologous regions from the alpha tetraploidy (panels 1 and 2), *Medicago trunculata* (panel 3), and the soybean homeologous regions from the beta tetraploidy event (panels 4 and 5). Circled is a gene in *Medicago* that has orthologs in all soybean homeologs except for soybean chromosome 1 (panel 1). (B) Diagram showing the homeologous sequences of soybean chromosome 1 (Glma01) and chromosome 2 (Glma02, panel 2). In chromosome 2 the circled gene from (A) (colored green in this diagram) is present, but absent in chromosome 1. Direct repeats (purple) and inverted repeats (blue) flank the sequence surrounding the gene in chromosome 2. Yellow denotes the syntenous sequence highlighted in pink from (A).
(0.18 MB PDF)

Dataset S6 **Generating the augmented sorghum gene list by comparison of sorghum to rice.** We used a pipeline to generate the sorghum gene list of SI1. Given the input of the same genomes and annotation, this pipeline generates this list repeatedly. This sorghum gene list includes the JGI official annotated sorghum genes plus the output of this pipeline: sorghum-rice ortholgous blastn hits that, when further analyzed, turned out to be homologous to RNA or protein-encoding genes or pseudogenes.
(0.76 MB PDF)

Dataset S7 **The script used to run the genetic algorithm for** **Figure 5** **.** The fitness of solutions in the evolutionary algorithm were scored using the Monte Carlo method as described in Methods (with the modification that rather than fixing the deletion length at 1 gene, deletion lengths were selected using the weighted averages generated by the evolutionary algorithm) with the most fit solutions being those where the median simulated number of deletion runs was least different from the observed number of runs. The genetic algorithm was allowed to run for 100,000 generations.
(0.17 MB PDF)
