# Supplementary material for: Following Tetraploidy in Maize, a Short Deletion Mechanism Removed Genes Preferentially from One of the Two Homeologs
Source: PLoS Biol. 2010 Jun 29;8(6):e1000409. doi: 10.1371/journal.pbio.1000409 (PMC2893956; doi:10.1371/journal.pbio.1000409)
Supplement: Dataset S2 — The sorghum-maize dot-plot. Sorghum (x-axis) and maize (y-axis) with alpha-tetraploidy lines colored purple by lower Ks from SynMap in CoGe. Numerals are chromosome numbers. Lower Ks is more recent. Although hundreds of breakpoints are evident, each segment of maize is orthologous to one sorghum region, and each sorghum segment is orthologous to two maize regions. (0.38 MB PDF) [file pbio.1000409.s002.pdf]

Supplemental Information 2

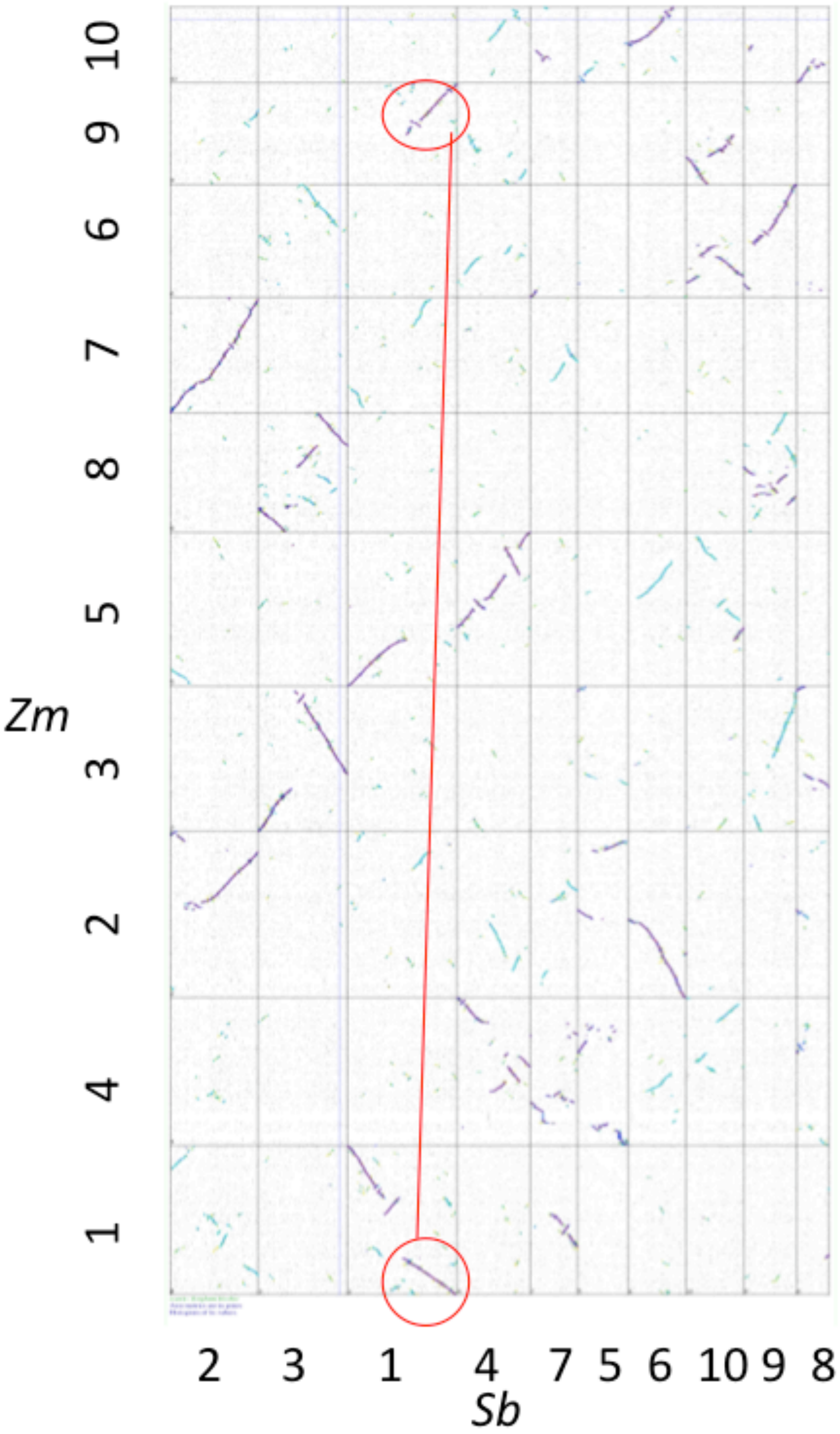

The *Sb* (x axis)-*Zm* (y axis) CDS=CDS dot-plot with alpha lines colored dark by lower Ks from SynMap in CoGe. Numerals are chromosome numbers. Lower Ks is more recent. Although 100s of breakpoints are evident, each segment of *Zm* is orthologous to one *Sb* region, and each *Sb* segment is orthologous to 2 *Zm* regions.

The 9 control *Sb*-*Zm*-*Zm* regions were chosen from this map. E.g. *Sb1* is illustrated: *Sb1*-=
proximal *Zm*1S(over)- *Zm*distal 9L (under)

****

Whole arms of maize that might be compared using map units. o= over-fractionated; u=under-fractionated
3L u=8L o perfect
2S u=10L o perfect
9L u=1S o perfect illustrated to left

DAGchainer settings –g,-D,-A= 10,20,5 genes; blastn
Sb genome in CoGe= 50X repeat masked from JGI Sbi1.4
Zm genome from maizegenome.org, 10-2009 release 50X repeat masked, gene filtered.

to regenerate a near-identical experiment using the SynMap application in CoGe, on-the-fly:
http://tinyurl.com/ygx2pu
