# Supplementary figures and images for: Following Tetraploidy in Maize, a Short Deletion Mechanism Removed Genes Preferentially from One of the Two Homeologs
Source: PLoS Biol. 2010 Jun 29;8(6):e1000409. doi: 10.1371/journal.pbio.1000409 (PMC2893956; doi:10.1371/journal.pbio.1000409)

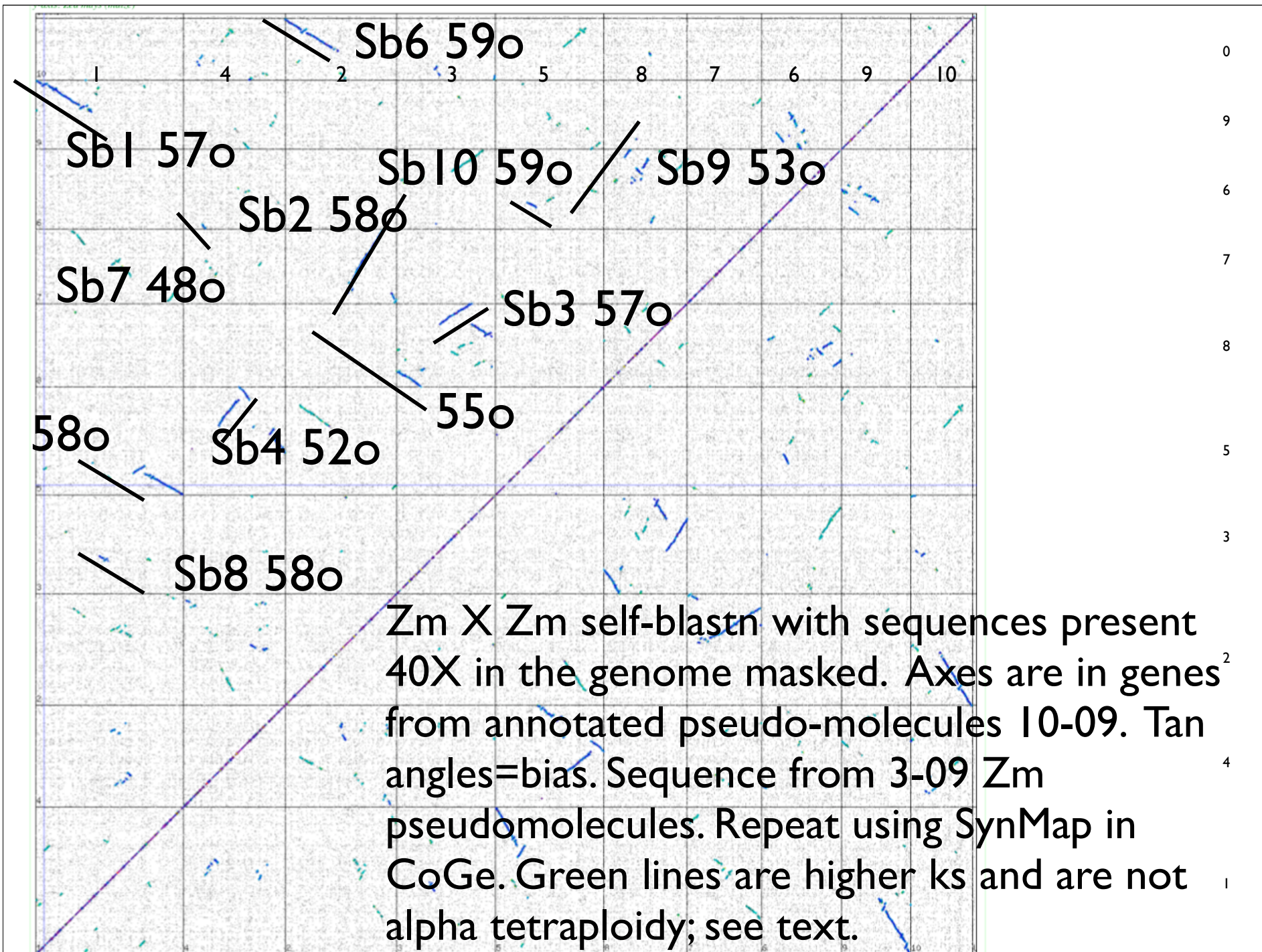

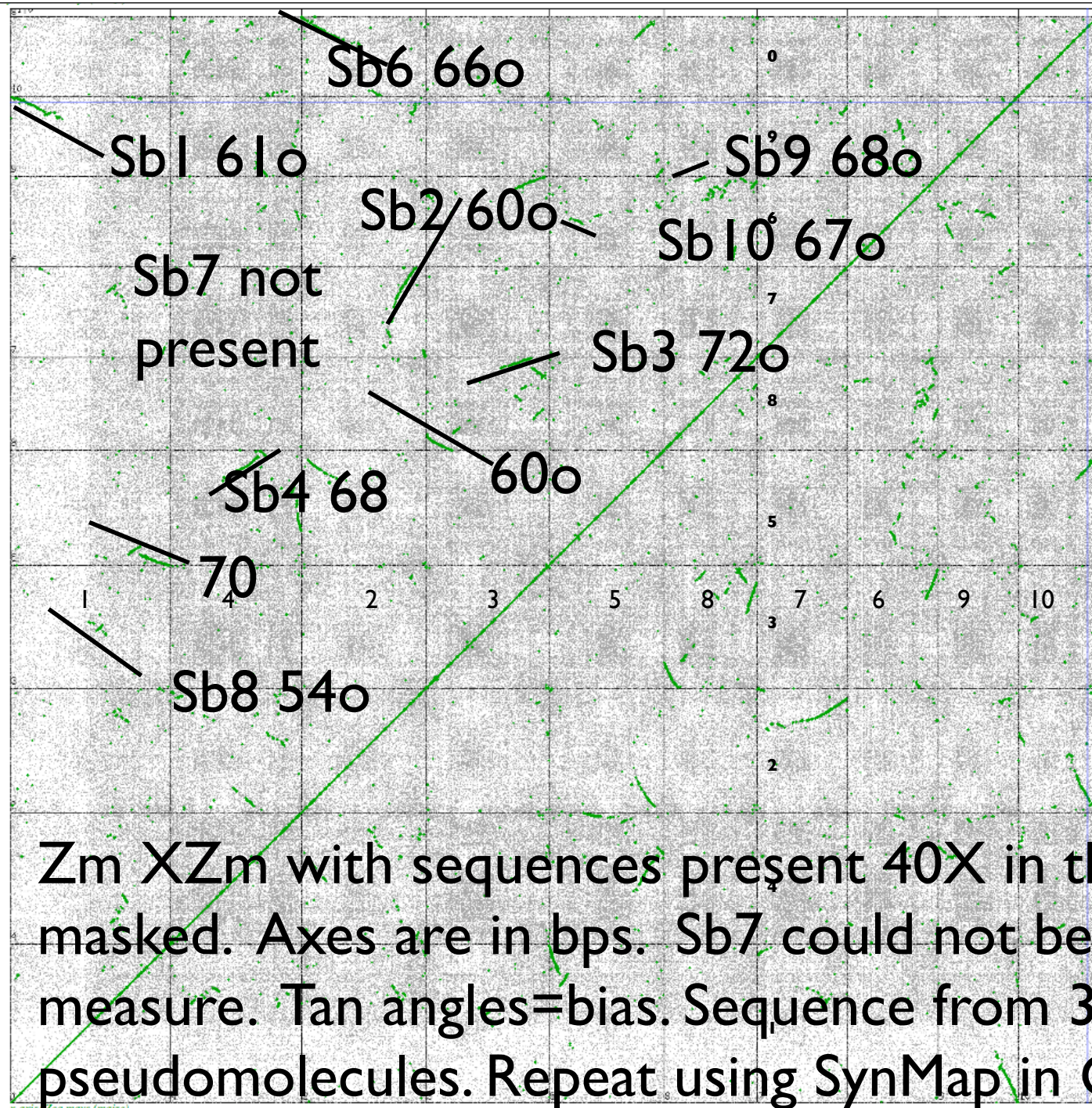

Supplement: Dataset S4 — Maize-maize self-blastn dot-plot. Sequences present 40×X in the genome were masked. Axes are in genes from annotated psudomolecules from 10-09. Tangent angles = bias. Green lines are higher Ks and are from the alpha-tetraploidy. (1.60 MB PDF) [file pbio.1000409.s004.pdf]
