# Supplementary material for: Following Tetraploidy in Maize, a Short Deletion Mechanism Removed Genes Preferentially from One of the Two Homeologs
Source: PLoS Biol. 2010 Jun 29;8(6):e1000409. doi: 10.1371/journal.pbio.1000409 (PMC2893956; doi:10.1371/journal.pbio.1000409)
Supplement: Dataset S5 — Whole-gene deletion in soybean ( Glycine max ). (A) A GEvo output of soybean homeologous regions from the alpha tetraploidy (panels 1 and 2), Medicago trunculata (panel 3), and the soybean homeologous regions from the beta tetraploidy event (panels 4 and 5). Circled is a gene in Medicago that has orthologs in all soybean homeologs except for soybean chromosome 1 (panel 1). (B) Diagram showing the homeologous sequences of soybean chromosome 1 (Glma01) and chromosome 2 (Glma02, panel 2). In chromosome 2 the circled gene from (A) (colored green in this diagram) is present, but absent in chromosome 1. Direct repeats (purple) and inverted repeats (blue) flank the sequence surrounding the gene in chromosome 2. Yellow denotes the syntenous sequence highlighted in pink from (A). (0.18 MB PDF) [file pbio.1000409.s005.pdf]

A

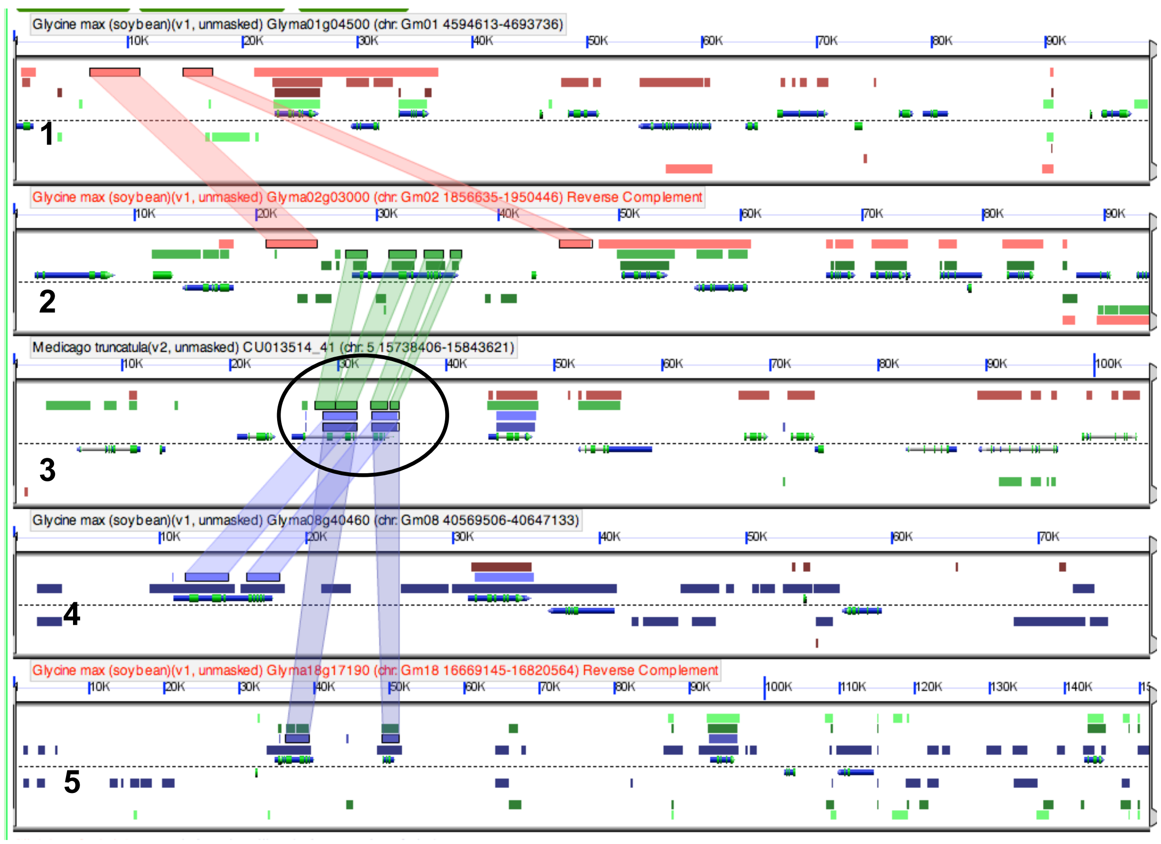

B

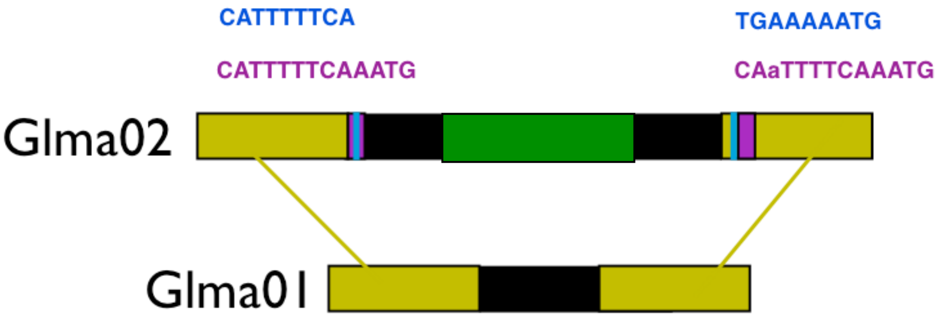

S5: Whole-gene deletion in soybean (*Glycine max*).
(A) A GEvo output of soybean homeologous regions from the alpha tetraploidy (panels 1 and 2), *Medicago truncatula* (panel 3), and the soybean homeologous regions from the beta tetraploidy event (panels 4 and 5). Circled is a gene in *Medicago* that has orthologs in all soybean homeologs except for soybean chromosome 1 (panel 1). (B) Diagram showing the homeologous sequences of soybean chromosome 1 (Glma01) and chromosome 2 (Glma02, panel 2). In chromosome 2 the circled gene from (A) (colored green in this diagram) is present, but absent in chromosome 1. Direct repeats (purple) and inverted repeats (blue) flank the sequence surrounding the gene in chromosome 2. Yellow denotes the syntenous sequence highlighted in pink from (A).
