# Supplementary material for: Following Tetraploidy in Maize, a Short Deletion Mechanism Removed Genes Preferentially from One of the Two Homeologs
Source: PLoS Biol. 2010 Jun 29;8(6):e1000409. doi: 10.1371/journal.pbio.1000409 (PMC2893956; doi:10.1371/journal.pbio.1000409)
Supplement: Dataset S6 — Generating the augmented sorghum gene list by comparison of sorghum to rice. We used a pipeline to generate the sorghum gene list of SI1. Given the input of the same genomes and annotation, this pipeline generates this list repeatedly. This sorghum gene list includes the JGI official annotated sorghum genes plus the output of this pipeline: sorghum-rice ortholgous blastn hits that, when further analyzed, turned out to be homologous to RNA or protein-encoding genes or pseudogenes. (0.76 MB PDF) [file pbio.1000409.s006.pdf]

Supplemental Information 6. Generating the augmented sorghum gene list by comparison of sorghum to rice. We used a pipline to generate the sorghum gene list of Supplemental Information 1. Given the input of the same genomes and annotation, this pipeline generates this list repeatedly. This sorghum gene list includes the JGI official annotated sorghum genes plus the output of this pipeline: sorghum-rice orthologous blastn hits that, when further analyzed, turned out to be homologous to RNA or protein-encoding genes or pseudogenes. Some of the genes we call using these comparative data were also present in the one or both maize orthologs. Only these "shared genes" were used in this study of fractionation. The genes that were called by this pipeline and not by JGI are of the form *sorghum_chromosomeX_ gene_start_stop*, not *SbxgXXXXXX*.

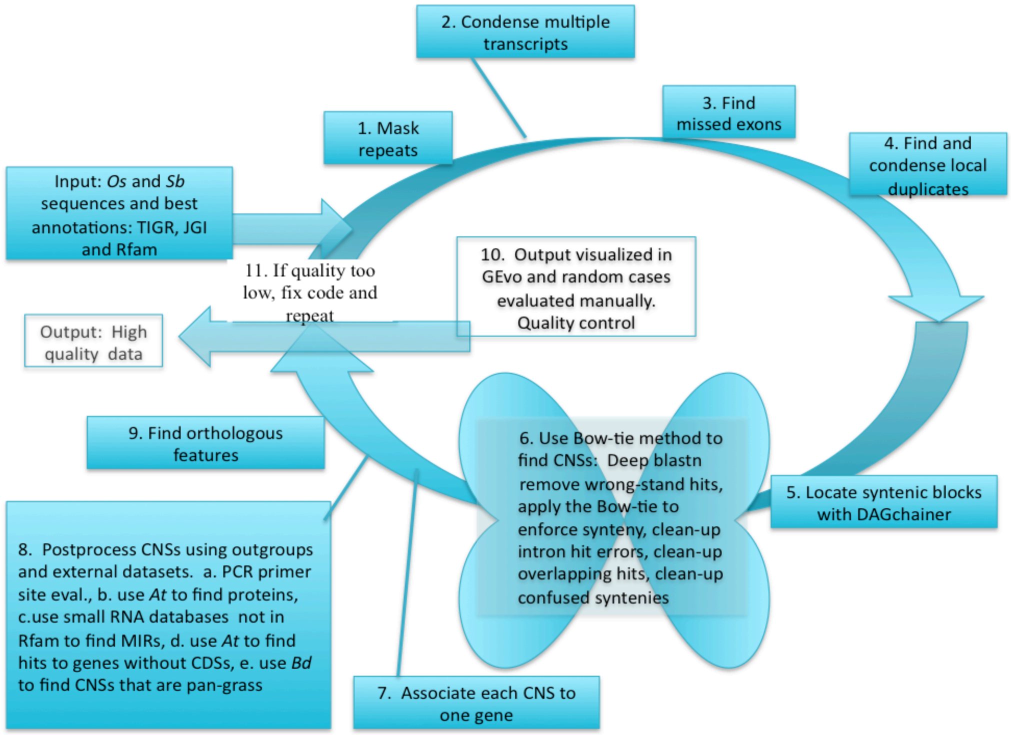

Figure 1. The CNS discovery pipeline is colored blue. In order to identify noncoding sequence, it is important to know as much as possible about coding sequence, so our pipeline generates alerts involving missed exons and new genes. BLAST (Altschul *et al.* 1990) was used throughout, and published applications (Hass *et al.* 2004, Rozin and Skaletsky 2000) were used in Steps 5 and 8. Steps 10 and 11 were used to develop the pipeline, but are not in the pipeline itself.

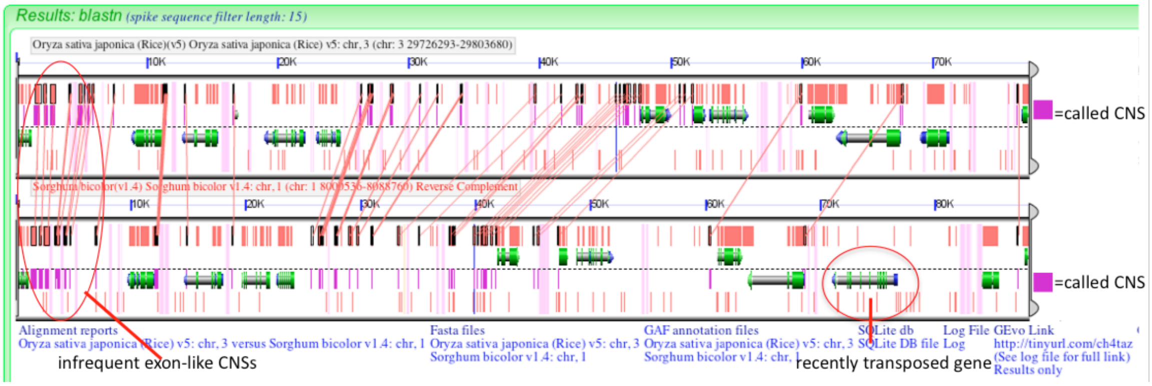

Figure 2. An 80-90 Kb segment of the *Os:Sb* orthologous chromosomes 3:4, where lines connect manually identified CNSs. These lines correspond with the purple blocks identifying Version1 CNSs pre-called by our pipeline. Exons are green; orange blocks are blastn high scoring pairs.

Experimental Procedures for the Pipeline (Brent Pedersen, Freeling lab, 10-2009)

Figure 1 above diagrams the automated, plant CNS discovery pipeline, Version 1. The input genomes of *Japonica* rice annotated by The Institute for Genomic Research (TIGR5) and Sbi1.4 sorghum sequence/annotation from the US DOE Joint Genomes Institute were downloaded from http://rice.plantbiology.msu.edu/pseudomolecules/info.shtml and http://www.phytozome.net/sorghum, respectively. The rice genome annotation was augmented by the addition of all *MIR* genes in Rfam, 2-5-09: http://rfam.sanger.ac.uk/genome/39947. During the evolution of this pipeline, the 10 steps detailed below cycled repeatedly until the quality of Version 1 was achieved. Steps 10 and 11 are skipped once a pipeline version is frozen.

Step 1. Mask repetitive sequences in input genomes. Chromosomal (pseudomolecule) sequence was masked for copy number following self-self blastn (Altschul, Gish, Miller,

Myers and Lipman 1990) at wordsize 15 (-W 15) and E-value < 0.001 (-e 0.001). Any base-pair (bp) position covered by a blast hit more than 50 times was masked with N and drawn in purple in our genomic viewer, GEvo (Genome Evolution; see Step 10). Similarly, any bp that was annotated as unsequenced is highlighted orange. We chose our blast settings to make sure that a test set of domesticated rice *Mu*-like transposons (each similar to TAIR Oryza Repeat DB3.1) were not removed: *Os04g36590, Os04g40060, Os03g15010, Os02g39540, Os03g62660, Os02g39520, Os03g45300,Os06g39680, Os08g06930, Os03g50900, Os02g34590, Os07g31400, Os07g37630, Os04g52560,Os03g56630, Os07g42400, Os03g08370, Os10g01550, Os06g07330, Os02g33460, Os04g33980,Os01g57230, Os04g54400, Os03g43990, Os12g41910, Os08g03650, Os03g52880, Os03g55830, Os12g02540, Os11g02620, Os03g22600, Os03g10880, Os02g35970, Os03g41350, Os12g39380, Os03g10800, Os06g08550,Os06g42640, Os02g09900* and *Os12g40530*; see Results.

Step 2. Condense multiple transcripts: Genes with alternative transcripts were condensed so all exons could be masked for CNS discovery. Splice variants were condensed into a single entity covering the union of all potential protein-coding sequences (CDSs). Condensation was done identically for mRNA or pre-RNA. Before finding CNSs, these unjoined, condensed features were masked, and the CNS-finding blast was run on the un-masked, noncoding sequence. A sequence for an mRNA-encoding gene, 'm,' with no annotated CDSs is not masked if it's hit overlaps a gene 'g' with any annotated CDSs. In this case, the sequence for 'm' is masked only up to the end of 'g.' This prevents the extremely rare case where 'm' would hide any CNSs in the UTR

of 'g.'

Step 3. Find missed exons/genes: In order to find putative missed exon annotations, each CDS sequence of rice was blasted against the entire genome sequence of sorghum--with called genes in the sorghum genome sequence masked. The lengths of resulting blast hits with an e-value < 0.01 to the same subject CDS sequence were summed, and hits in groups with a sum of greater than 100 were recorded as a missed exon. These hits were given a name based on the organism, chromosome, and base-pair location.

This was repeated for sorghum genes blasted against the 50X repeat masked rice genome sequence. Hits to subject sequence where the gene containing the query CDS is not paired and the query CDS is not a local duplication are designated unbalanced pairs. The subject homeolog of the unbalanced pair was either added to an existing annotation (data accumulated in separate GFF sheets as Supplemental Tables 2a/b) or called as a new gene and given a name like "rice chromosome_start-stop" New genes are listed on the genes list for both rice and sorghum. Note: TIGR-like LOC#s are used only for officially annotated genes.

The unannotated subject HSP was added to an existing annotation if the annotated subject exon hit the same query feature as the unannotated HSP, or a subject annotation is flanked on both sides by subject HSPs that hit a single query annotation. Multiple unannotated HSPs in the same area were condensed into a single new rice gene designation if they all hit the same query gene.

Step 4. Condense local (tandem) duplications. For each of rice and sorghum, annotated

CDS, gene, tRNA, and mRNA sequences were saved into fasta files and each chromosome was blasted to self at the same parameters as in Step 1 above. Using our local duplication finder we found local duplications using the following rules: 3a. Genes are numbered by chromosomal order and given integer positions on each chromosome. 3b. Any blast hit of gene A with integer order Ai to another gene B with order Bi where abs(Ai - Bi) ≤ 4 was considered a set of local duplications. 3c. If A and B were in a local duplicate set, given another gene C with position Ci such that either (Ai - Ci) ≤ 4 OR (Bi - Ci) ≤ 4, Ci is added to that set of local duplicates. 3d. The lowest ordered gene was always arbitrarily assigned as the parent of the duplicates, with the other duplicates being the arbitrary daughters. Because of rule 3c, a tandem array can extend out to 20 or more genes, as long as each gene order is no more than 4 away from its closest neighbor. Only the parent duplicates were used in the remaining analyses. CNS behavior during and after tandem duplication was not studied here.

Step 5. Locate syntenic blocks: DAGchainer (Hass, Delcher, Wortman and Saltzberg 2004) was used to find segmental duplications between sorghum and rice. CDS sequence was used if available. If not, then the TIGR annotation "gene" and then the mRNA sequences were used. The blast output of E value < 0.001 was prefiltered with the filter_repetitive_matches.pl script provided and documented with DAGchainer, using a window size of 100000. DAGchainer was run with default parameters except for " -g 32000 -D 160000." Finally, a custom script was used to search along the DAGchainer-found diagonals and to add in any syntenic genes that occurred within 80,000 bps of a diagonal. Since DAGchainer finds only the "best" path through a set of potential

diagonals, it is expected to miss some syntenic pairs. This post-processing adds new pairs into our set before CNSs are discovered.

Step 6. Find CNSs using the Bow-tie method: The 50X repeat-masked genome was masked further. Any CDS, gene, or mRNA sequence was replaced with 'N's, leaving only nonrepetitive, noncoding sequence (including introns) in which to find CNS's. For each pair of syntenous genes, the following steps were repeated:

6a. Deep blastn. The masked sequence of each rice homolog, including 12000 bps up- and down-stream, was blasted (bl2seq) against its sorghum pair using parameters: "-e 2.11 -W 7 -Y 812045000." Setting these constant make the e-values of the resultant blast output invariant with the size of the query and subject sequences. In addition, these exact values simulate the probability of finding a 15/15 exact match--about the noise level for finding plant CNSs (Kaplinsky *et al*. 2002, Lyons and Freeling. 2008). The following steps filtered these blast HSPs (high scoring pairs) leaving only spatially valid CNSs.

6b. Remove wrong-strand hits. Any CNS blast hits with a different orientation than the homologous pair were removed.

6c. The Bow-tie. A Bow-tie was created in x-y space to enforce synteny. The Bow-tie is an expanding polygon-- shaped like its namesake-- extending from either end of the gene-pair in 2-D space--a space traditionally viewed as a dot plot. Dots (blastn HSPs) falling in that region were found using a simple point-in-polygon routine: e.g. http://www.ecse.rpi.edu/Homepages/wrf/Research/Short_Notes/pnpoly.html. We used a geographic library GEOS (http://trac.osgeo.org/geos/) with the python bindings provided by Shapely (http://trac.gispython.org/lab/wiki/Shapely) for these queries as they also

provide the methods to see if syntenic lines cross or if shapes overlap; we used both operations to pare-down CNSs from the pool of candidates in the Bow-tie. For example, a CNS homolog 100 bps upstream from the x-gene, must have its pair about 1000 bps upstream from the y-gene. For CNSs farther up/downstream, this difference is less stringent, allowing for small insertions and deletions while retaining synteny. Any CNS falling outside of this bow-tie was removed.

6d. Intron clean-up, part1. Any putative CNS in the intron of one homolog, but not the other, is removed.

6e. Intron cleanup, part2. Any putative CNS in the intron of any upstream gene on either homeolog was removed from that Bow-tie. However, if the removed CNS occurred within the Bow-tie of another syntenic pair, it would still be considered when the bow-tie was run over that pair.

6f. Overlapping CNS cleanup. For CNS's that overlapped, from each group with overlaps, the CNS pair with the highest e-value was removed until there were no more overlaps.

6g. Confused synteny cleanup. For any CNSs that were out of synteny, meaning the lines connecting syntenic features crossed, we first removed any HSP creating the endpoints of a line that crossed more than 3 others. With the remaining crossing groups, CNSs were removed in order of descending e-value until all crossed syntenic lines were removed.

Step 7. Associate each CNS to one gene: Each CNS was assigned to a nearby orthologous gene. The CNS's were sorted first by the number of intervening retained

genes, then by distance. A CNS was removed if the closest sorted gene had any intervening *paired* genes; unpaired genes, like transposon insertions, were skipped. Usually, CNSs clustered around a single gene. In reality, any one CNS could act on any adjacent gene or genes.

Step 8. Post process using outgroups and external datasets: Information on each putative CNS was compiled.

8a. CNS as PCR primer site evaluation. Each CNS sequence with length≥18 was checked using Primer3 (Rozin and Skaletsky 2000) for being a quality PCR site. Any hit for which Primer3 found the same primer sequence for both query and subject ortholog was classified as "A." CNS pairs were classified as a "B" primer sites if Primer3 found a PCR sequence in one ortholog and the 5 bps at the 3'-most end were found at the same location in the other ortholog, where "same location" allowed a 2 bp difference in location. Finally, any remaining CNS for which Primer3 found a PCR site in only a single ortholog of the pair was classified as 'C'. Any CNS classified as PCR A, B, or C was included with the nearest gene occurring to the left of (lower base-pair position) each ortholog.

8b. Find CNSs that are really proteins using the At *outgroup.* All CNSs of length ≥18 were blastx'ed against Arabidopsis (TAIR8) proteins. Any CNS with a hit to *At* with an e-value < 0.01 *and* a bitscore ≥ 50 was classified as a protein coding. Also, any CNS with a blast hit to *At* with a bitscore of ≥ 45 and a coverage of ≥ 90% was labeled as protein coding. The TAIR8 description of the best hit was reported. Those CNSs that match TAIR8 proteins were reassigned as new genes/pseudogenes.

8c. Find putative CNSs that match small RNAs not already in Rfam. CNSs of length ≥ 18 were compared for exact matches to a list of known miRNAs and smRNAs from the Sundarasan lab website, UC-Davis, USA on 3-20-09: http://www-plb.ucdavis.edu/Labs/sundar/members.htm# (click "databases.") Any CNS with an exact match to a *MIR* miRNAs was redesign Ted a probable *MIR* gene, but matches to siRNAs were simply recorded on Supplemental Table 3.

8d. Finding putative CNSs that probably encode/encoded an RNA. All CNSs were blastn'ed to 50X repeat-masked sequence of Arabidopsis RNAs (tRNA, snoRNA, siRNA, miRNA). Any Arabidopsis accession from ftp://ftp.arabidopsis.org/home/tair/Genes/TAIR7_genome_release/TAIR7_gff3/TAIR7_GFF3_genes.gff that didn't have a CDS was used in this database, including pseudogenes. The BLAST parameters used were: -e 0.001 -m 8 -W 7. For each CNS in the blast output, we report the best Arabidopsis hit and its TAIR7 description (ftp://ftp.arabidopsis.org/home/tair/Genes/TAIR7_genome_release/TAIR7_functional_descriptions)

8e. Use the genome of Brachypodium distachyon (Bd) *to make a pan-grass assessment*. The initial release of the *Bd* genome (DOE JGI) was downloaded from http://www.brachypodium.org/downloads . Both the query and subject sequence of each CNS was blastn'ed to the full *Bd* sequence using artificial cutoff with parameters: -p blastn -e 0.1 -F F -E 2 -G 5 -q -2 -Y 812045000 -m 8 -W 7. The blast hits were then limited to those that were within 15,000 bp of the gene of the corresponding *Os* or *Sb* gene. This was a simple way to enforce 1-gene synteny and remove obviously spurious hits. For each CNS, all remaining blast hits were sorted by e-value. The hits were iterated

pairwise until a difference of 10-fold was found between the adjacent hits. Only those hits above the 10-fold gap were included in the final count. This provided an approximation of the number of quality, potentially syntenic hits of a CNS in *Bd*.

Step 9. Find orthologous features. Each feature -- each part of some syntenic region— is either "orthologous" or "deeper." A deeper syntenous block might be a sorghum chromosomal stretch paired with a rice chromosomal segment derived from the homeologous chromosome to the ortholog expected following the pre-grass tetraploidy. We report here on the orthologous *Os*-*Sb* features only. This sorting is done by consulting an orthologies list prepared by MF manually using best blast hit lists, in the format: *Os* chr., *Sb* chr., *Os* start, *Os* stop, *Sb* start, *Sb* stop, where 999999999= maximum bps.

1,3,0,999999999,0,9999999999
2,4,0,999999999,0,9999999999
3,1,0,999999999,0,999999999
4,6,0,999999999,0,999999999
5,9,0,999999999,0,999999999
6,10,0,999999999,0,999999999
7,2,0,999999999,0,999999999
8,7,0,999999999,0,999999999
9,2,0,999999999,0,999999999
10,1,0,999999999,17142672,53887670
11,5,0,999999999,0,999999999
12,8,0,999999999,0,999999999

This orthologies list is identical to that published in the sorghum launch paper (Paterson *et al*. 2009). After freezing, we found out that simple, manual curation of orthologous genes is still necessary for our Version 1 gene lists (Supplemental Tables 1 and 2) because, for a few genes only, an orthologous syntenic line and a sparser syntenic line reflecting deeper homologies exist for the same *Os*-*Sb* gene pair. This infrequent ambiguity will be fixed in Version 2 of our pipeline. This is important because genes with two orthologs may have a slightly inflated orthologous CNS counts.

Step 10. Visual proofing of pipeline output. Without a way to test orthologous regions of *Os-Sb* for how annotation error and evolutionary randomness affected automated CNS calls, we could not have achieved quality output from such biologically complex input. Our output has a "GEvo link" for each row of data. Clicking this link allows for proofing each feature, be it a gene or a putative CNS, and the *Os-Sb* syntenic chromosomal regions around them. GEvo is the alignment graphic research tool within the comparative genomics package called "CoGe" (Lyons andFreeling 2008, Lyons *et al*.2008). To facilitate proofing, the CNS calls following each run of the pipeline were added to the official gff annotation database and, when this test database is chosen, these CNS calls were drawn on the model line of GEvo as a purple rectangle. The link automatically pulls these test databases for display in GEvo. Then actual CNSs can be computed on-the-fly and compared with those CNS derived from this pipeline. Errors were apparent, and suggested coding fixes. Figure 2 of this Supplemental Information is a screenshot of such a proofing panel in GEvo after considerable debugging. Visual proofing is an important and essential step in our pipeline methods, even though it is not in the pipeline itself.

Step 11. Repeat until error rate is acceptable. This pipeline was repeated until an error rate below 5% was achieved. Just over 200 CNSs were proofed from approximately 50 genes chosen approximately at random over the paired genomes. Even so, missed CNSs do exist. In particular, exons can be missed near mis-annotated exons.

Ahn, S. and Tanksley, S.D. (1993) Comparative linkage maps of the rice and maize genomes. *Proc Natl Acad Sci U S A*, 90, 7980-7984.

Altschul, S.F., Gish, W., Miller, W., Myers, E.W. and Lipman, D.J. (1990) Basic local alignment search tool. *J Mol Biol*, 215, 403-410.

Bennetzen, J.L. (2007) Patterns in grass genome evolution. *Curr Opin Plant Biol*, 10, 176-181.

Castellana, N., Payne, Z., Stanke, M. and Bafna, V., et. al (2009) Proteogenomic discovery, correction and confirmation of Arabidopsis gene models. *PNAS*, in press.

Freeling, M. (2009) Bias in plant gene content following different sorts of duplication: tandem, whole-genome, segmental, or by transposition. . *Annual Review of Plant Biology*, 60, 433-453.

Freeling, M., Lyons, E., Pedersen, B., Alam, M., Ming, R. and Lisch, D. (2008) Many or most genes in Arabidopsis transposed after the origin of the order Brassicales. *Genome Res*, 18, 1924-1937.

Freeling, M., Rapaka, L., Lyons, E., Pedersen, B. and Thomas, B.C. (2007) G-boxes, Bigfoot genes and environmental response: characterization of the intragenomic conserved noncoding sequences of Arabidopsis. . *The Plant Cell*, 19.

Freeling, M. and Subramaniam, S. (2009) Conserved noncoding sequences (CNSs) in higher plants. *Curr Opin Plant Biol*, 12, 126-132.

Gale, M.D. and Devos, K.M. (1998) Comparative genetics in the grasses. *Proc Natl Acad Sci U S A*, 95, 1971-1974.

Guo, H. and Moose, S.P. (2003) Conserved noncoding sequences among cultivated cereal genomes identify candidate regulatory sequence elements and patterns of promoter evolution. *Plant Cell*, 15, 1143-1158.

Hass, B., Delcher, A., Wortman, J. and Saltzberg, S. (2004) DAGChainer: a tool for mining segmental genome duplications and synteny. . *Bioinformatics*, 20, 3643-3646.

Inada, D.C., Bashir, A., Lee, C., Thomas, B.C., Ko, C., Goff, S.A. and Freeling, M. (2003) Conserved noncoding sequences in the grasses. *Genome Res*, 13, 2030-2041.

Kaplinsky, N.J., Braun, D.M., Penterman, J., Goff, S.A. and Freeling, M. (2002) Utility and distribution of conserved noncoding sequences in the grasses. *Proc Natl Acad Sci U S A*, 99, 6147-6151.

Kellogg, E.A. (2001) Evolutionary history of the grasses. *Plant Physiology*, 125, 1198-1205.

Lyons, E. and Freeling, M. (2008) How to usefully compare homologous plant genes and chromosomes as DNA sequence. *The Plant Journal*, 53, 661-673.

Lyons, E., Pedersen, B., Kane, J., Alam, M., Ming, R., Tang, H., Wang, X., Bowers, J., Paterson, A., Lisch, D. and Freeling, M. (2008) Finding and comparing syntenic regions among Arabidopsis and the outgroups papaya, poplar, and grape: CoGe with rosids. *Plant Physiol*, 148, 1772-1781.

Moore, G., Devos, K.M., Wang, Z. and Gale, M.D. (1995) Cereal genome evolution. Grasses, line up and form a circle. *Curr Biol*, 5, 737-739.
Nelson, R.J., Naylor, R.L. and Jahn, M.M. (2004) The role of genomic research in the improvement of "orphan" crops. *Crop Sciences*, 44, 1901-1904.
Paterson, A.H., Bowers, J.E., Bruggmann, R., Dubchak, I., Grimwood, J., Gundlach, H., Haberer, G., Hellsten, U., Mitros, T., Poliakov, A., Schmutz, J., Spannagl, M., Tang, H., Wang, X., Wicker, T., Bharti, A.K., Chapman, J., Feltus, F.A., Gowik, U., Grigoriev, I.V., Lyons, E., Maher, C.A., Martis, M., Narechania, A., Otillar, R.P., Penning, B.W., Salamov, A.A., Wang, Y., Zhang, L., Carpita, N.C., Freeling, M., Gingle, A.R., Hash, C.T., Keller, B., Klein, P., Kresovich, S., McCann, M.C., Ming, R., Peterson, D.G., Mehboob ur, R., Ware, D., Westhoff, P., Mayer, K.F., Messing, J. and Rokhsar, D.S. (2009) The Sorghum bicolor genome and the diversification of grasses. *Nature*, 457, 551-556.
Prasad, V., Stromberg, C. A. E. , Alimohammadian, H., Sahi, A. (2005) Dinosaur coprolites and the early evolution of grasses and grazers. *Science*, 310, 1177-1180.
Rozin, S. and Skaletsky, H. (2000) Primer3 on the WWW for general users and for biologist programmers. In *Bioinformatics: Methods and protocols: Methods in Molecular Biology* (Krawetz, S. and Misener, S. eds). Totowa, NJ: Humana Press, pp. 365-386.
Salvi, S., Sponza, G., Morgante, M., Tomes, D. and niu, X.e.a. (2007) Conserved noncoding genomic sequences associated with a flowering-time quantitative trait locus in maize. *PNAS USA*, 104, 11376-11381.
Tang, H., Bowers, J.E., Wang, X., Ming, R., Alam, M. and Paterson, A.H. (2008) Synteny and collinearity in plant genomes. *Science*, 320, 486-488.
Thomas, B.C., Rapaka, L., Lyons, E., Pedersen, B. and Freeling, M. (2007) Intragenomic conserved noncoding sequences in Arabidopsis. *Proc. Natl. Acad. Sci.*, 104, 3348-3353.
