# Supplementary material for: Following Tetraploidy in Maize, a Short Deletion Mechanism Removed Genes Preferentially from One of the Two Homeologs
Source: PLoS Biol. 2010 Jun 29;8(6):e1000409. doi: 10.1371/journal.pbio.1000409 (PMC2893956; doi:10.1371/journal.pbio.1000409)
Supplement: Dataset S7 — The script used to run the genetic algorithm for Figure 5 . The fitness of solutions in the evolutionary algorithm were scored using the Monte Carlo method as described in Methods (with the modification that rather than fixing the deletion length at 1 gene, deletion lengths were selected using the weighted averages generated by the evolutionary algorithm) with the most fit solutions being those where the median simulated number of deletion runs was least different from the observed number of runs. The genetic algorithm was allowed to run for 100,000 generations. (0.17 MB PDF) [file pbio.1000409.s007.pdf]

```
"""
This module does operations on data that looks like:
    >>> runs_data = '1__1111_11_1__111___111_11111'

where '1' indicates a gene retention and '_', a gene loss.
that can be converted by count_runs to a runs list like:
    >>> count_runs(runs_data)
    [(1, 2), (2, 1), (3, 2), (4, 1), (5, 1)]

indicating 2 runs of 1, 1 run of 2, 2 runs of 3, etc.

the run_sim() function takes a string as in runs_data above
and runs a genetic algorithm to find the deletion length
frequencies that best reproduce it's pattern.

it does so by calling the `gen_deletions` function, which
takes a list of deletion lengths and a string of '1's and
randomly chooses a deletion length from the list of deletion
lengths and a point to start deleting. this continues until
the simulated string contains as meny deletions as the observed
string. then the runs are counted with count_runs()
and the numbers are compared to the runs data of the observed
string. the GA tries to minimize the difference between
the run counts.

"""
import sys

from pyevolve import G1DList
from pyevolve import GSimpleGA
from pyevolve.Consts import minimaxType

from itertools import groupby
from random import randint, choice
try:
    import psyco
    psyco.full()
    print >>sys.stderr, "using psyco"
except:
    print >>sys.stderr, "no psyco"

def count_deletion_runs(astr, deletion="_"):
    """
    like count runs except it counts the run-lengths
    of deletions, not retentions
    `astr` is a string where anything that is not `deletion`
    is considered to be retained.
    """
    delstr = list(set(astr).difference(deletion))
    assert len(delstr) == 1, delstr
    delstr = delstr[0]
    return count_runs(astr, splitter=delstr)

def count_runs(astr, splitter="_"):
    """find the length of runs in the str
    ::

```

```
        >>> count_runs("11_111_1_1_1_1_111111")
        [(1, 4), (2, 1), (3, 1), (4, 0), (5, 0), (6, 1)]

    """
    lens = sorted([len(s) for s in astr.split(splitter) if s != ''])
    nmax = lens[-1]
    runs = dict((x, len(list(y))) for (x, y) in groupby(lens, lambda a: a))
    for i in range(1, nmax + 1):
        # if it's not there, set it.
        runs.setdefault(i, 0)
    return sorted(runs.items())

def gen_deletions(region_length, deletion_lengths, num_deletions=0.5,
                  count_retentions=False):
    """
    :param region_length: the length in genes of the region to simulate
    :param deletion_lengths`: an iterable of numbers to choose randomly for the
                        number of genes to delete
    :param num_deletions: if > 1, then the number of times to do a deletion event
                        if < 1, then the proportion of the region to delete before
                        stopping
    :param count_retentions: by default (False), this will count the runs of deletions
                              if True, it will count runs of retention.
    :rtype: the generated string and the run counts and the number of deletion
            events. (see count_runs in misc.py)
    """
    if num_deletions < 1:
        num_deletions = int(round(num_deletions * region_length ))

    region = range(region_length)
    deletion_count = 1
    current_region_length = region_length

    for event in range(num_deletions):

        # can be: randint(0, region_length - 1 - deletion_count)
        #deletion_start = randint(0, len(region) - 1)
        #assert current_region_length == len(region), (current_region_length, len(region), event)
        deletion_start = randint(0, current_region_length - 1)

        deletion_length = choice(deletion_lengths)

        deletion_stop = deletion_start + deletion_length
        if deletion_stop > current_region_length:
            deletion_length -= (deletion_stop - current_region_length)
            deletion_stop = current_region_length



        del region[deletion_start:deletion_stop]
        current_region_length -= deletion_length


        deletion_count += deletion_length
        if deletion_count > num_deletions:
            break

    region = frozenset(region)
    deletion_string = "".join(["1" if i in region else "_" \
                                 for i in range(region_length)])

    if deletion_lengths == (1,):
        assert len(deletion_string) == region_length, (len(deletion_string), )

    if count_retentions:
        runs = count_runs(deletion_string)
    else:
        runs = count_runs(deletion_string, "1")

    return deletion_string, runs

def initializator(genome, **args):
```

```
    """ used for the genetic algorithm initialize the deletion lengths
    to randomly chosen values between 1 and 6"""
    genome.clearList()
    rmin = genome.getParam("rangemin")
    rmax = genome.getParam("rangemax")
    for i in range(GA_LEN):
        genome.append(randint(rmin, rmax))

def mutator(genome, **args):
    """
    used for the genetic algorithm. mutate a "chromosome" by
    picking between 0 and 4 mutations in random spots and incrementing
    or decrementing by 1.
    """
    rmax = genome.getParam("rangemax")
    rmin = genome.getParam("rangemin")
    #l = len(genome)
    l = GA_LEN
    n_mutations = randint(0, 4)
    mutations = 0
    while mutations < n_mutations:
        idx = randint(0, l - 1)
        change = choice([-1, 1])
        v = genome[idx]
        newval = v + change

        if newval > rmax: newval -= 2
        if newval < rmin: newval += 2

        genome[idx] = newval
        mutations += 1
    return mutations


def run_sim(astr):
    """
    given an example deletion/retention string, where
    "_" is a deletion, run a ga simulation to determine
    the deletion lengths likely to have created that pattern
    of deletion-lengths
    """

    num_deletions = astr.count('_')
    region_length = len(astr)


    real_runs = count_deletion_runs(astr)
    max_real_run_len = real_runs[-1][0]

    def evaluator(chromosome):
        deletion_lengths = list(chromosome)

        # since gen_deletions is random, do multiple tries to
        # make sure an outlier doesnt screw it up.
        ntries = 10
        asum = 0.0
        for tries in range(ntries):
            sim_str, sim_runs = gen_deletions(region_length,
                                              deletion_lengths=deletion_lengths,
                                              num_deletions=num_deletions,
                                              count_retentions=False)
            sim_runs = dict(sim_runs)

            for run_length, real_count in real_runs:
                asum += run_length * abs(real_count - sim_runs.get(run_length, 0))
            # maybe the simulation had some really long runs...
            for run_length in range(max_real_run_len + 1, max_real_run_len + 10):
                asum += run_length * sim_runs.get(run_length, 0)
        return asum / ntries


    genome = G1DList.G1DList(len(astr))
```

```
    # deletion lenghts vary between 1 and 5
    genome.setParams(rangemin=1, rangemax=5, roundDecimal=5)
    genome.initializator.set(initializator)
    genome.mutator.set(mutator)
    genome.evaluator.set(evaluator)

    ga = GSimpleGA.GSimpleGA(genome)
    ga.setMinimax(minimaxType['minimize'])
    ga.setGenerations(GA_GENERATIONS)
    ga.evolve(freq_stats=0)
    best = ga.bestIndividual()
    return {'deletion_lengths': sorted(list(best)), 'fitness': best.fitness,
            'score': best.score}

GA_LEN = 25
GA_GENERATIONS = 10000
MAX_DELETION_SIZE = 10 # > 1000 is same as no removal.

if __name__ == "__main__":
    # expects a string of deletions 1_1111_11 where "_" is the deletion
    # and a number which is the max deletions to allow (more are removed)
    import sys
    if len(sys.argv) > 1:
        print "testing..."
        import doctest
        doctest.testmod()
        sys.exit()


    import re
    delstrs = dict(
        #overs = open('over.txt').read().strip(),
        #unders = open('under.txt').read().strip(),
        both = open('both.txt').read().strip()
    )
    # all deletions longer than this are collapsed to nothing
    # assumed not to be real.
    print "removing deletions longer than % i" % MAX_DELETION_SIZE
    for delname, deletion_str in delstrs.items():
        deletion_str = re.sub("_{%i,}" % MAX_DELETION_SIZE, "", deletion_str)
        print
        print delname
        print run_sim(deletion_str)
```

©2009 Google - Code Home - Terms of Service - Privacy Policy - Site Directory - Project Hosting Help

Hosted by Google code
